# Guard dog behaviour (*Canis lupus familiaris*) towards various animal species and humans on farms in Germany

**DOI:** 10.1371/journal.pone.0337432

**Published:** 2025-11-25

**Authors:** Konstanze Krueger, Kimberly Scarlet Camenzind, Aida Kumpf, Kate Farmer, Maren Bernau

**Affiliations:** 1 Faculty of Agriculture, Economics and Management, Nuertingen-Geislingen University, Nürtingen, Germany; 2 Zoology/Evolutionary Biology, University of Regensburg, Regensburg, Germany; 3 Centre for Social Learning and Cognitive Evolution, School of Psychology, University of St Andrews, St Andrews, United Kingdom; UFSJ: Universidade Federal de Sao Joao del-Rei, BRAZIL

## Abstract

Over recent decades, several predator species have returned to human-dominated landscapes in Europe, with wolves (*Canis lupus)* causing the most damage to livestock. In Germany, some ‘pioneer’ farmers started keeping guard dogs (*Canis lupus familiaris)* to protect their livestock, but these ‘pioneers’ faced opposition from a general public unfamiliar with methods of protecting against predators. To evaluate the use and management of guard dogs to protect various farm animal species against predators in rural areas frequently used by the public in Germany, we studied the behaviour of 113 guard dogs on farms across Germany that have frequent public contact. Two approaches were used: I) we observed guard dog proximity to and behaviour towards goats and horses with direct field observation, and II) we asked equine science and agriculture students trained in behaviour observations and official herd management commissioners to report their experiences of guard dogs during their initial visits to farms keeping various livestock species. These reports included observations of the dogs’ behaviour and information about the farm and dog management practices, and showed that guard dogs preferentially stay within 1 farm-animal body lengths of goats and horses. They adapted to a large variety of tasks and could protect various species. They displayed friendly behaviour towards the owners of the farms and known persons, and all behaviour categories towards farm animals and unfamiliar persons in the presence of the owner. They were dominant and watchful towards unknown persons and external dogs. The farmers’ training and socialising of their guard dogs appear to be successful, as older dogs, and large mixed-sex guard dog groups were consistently watchful against external individuals, but friendly towards farm personnel. In conclusion, guard dogs adapt well to guarding various species on German farms.

## Introduction

Over the past few decades, several predator species, such as common ravens (*Corvus corax*) [1], golden jackals (*Canis aureus*) [2], wolves (*Canis lupus*) [3,4], brown bears (*Ursus arctos*) [5], and, even though recently stagnating, lynx (*Lynx lynx*) [6], have made a return to human-dominated landscapes in Europe [3,4,7,8]. Therefore, European farmers have been increasingly confronted with the need to consider livestock protection measures, and guard dogs (*Canis lupus familiaris)* have been described as a particularly effective protection against predators [9,10]. In Germany, some ‘pioneer’ farmers have started keeping guard dogs for the protection of various farm animal species. However, these pioneers face opposition from a society with little understanding of livestock protection measures or of how to deal with the presence of guard dogs [11,12]. This study sets out to evaluate the use and management of guard dogs when protecting various farm animal species against predators in rural areas highly frequented by the public in Germany.

The predator species causing most damage in livestock in Germany are wolves (*Canis lupus*) [13]. Although now downregulated to a protected status, wolves were strictly legally protected in the European Union from 1992 to 2025 [14,15]. From around the year 2000, wolves began recolonizing Germany from the northeast, while a few also entered the region by crossing the Alps from Italy [16]. The wolf population in Germany reached 185 wolf packs, 45 wolf pairs and 22 solitary, territorial animals in 2023 [13], with the highest density in the north-eastern counties of Saxony, Lower-Saxony and Brandenburg, and the lowest in the southern states of Bavaria and Baden-Württemberg. The recovery of the wolf population was warmly welcomed by some parties [12,17–19]. Aside from the symbolic meaning of wolves [17,19], it was also argued that the biodiversity of strictly protected nature conservation areas in Germany would benefit from large predators, such as wolves [20]. This was demonstrated, for example, with a wolf recolonization project in the Yellowstone National Park in the USA [21], where wolves reduced overpopulations of wild boar, deer, rabbits, and other animal species [21], and limited the spread of animal diseases, such as African swine fever [20].

However, an increase of 12% in wolf attacks and 31% in damage done to livestock by wolves, was reported in Germany in 2023 [13]. The 5727 reported cases comprised 4957 sheep, 174 goats, 234 cattle, 315 managed deer, and 47 further animals, of which 10 were domestic dogs, 8 were alpacas and 29 were equids, comprising 4 donkeys and 25 horses [13]. Therefore, as in several other European countries, German farmers were advised, and given financial support, to implement predator protection measures [22,23], including buying and keeping guard dogs (see for details: [13]). Integrating guard dogs into livestock groups improved the coherence of the groups and diminished the risk of animals being captured by predators [10]. Furthermore, the presence of guard dogs reduced fear and stress and increased the productivity of the guarded animals, and this, in turn, had a positive impact on the farmers [24].

Studies on guard dogs in the USA [10], Canada [25], Switzerland [26], and Australia [9,24,27] have established the background to their deployment in these regions. Guard dogs are exclusively bred to live with and protect a wide range of farm animal species, from small animals, e.g., poultry, sheep and goats, up to cattle and equids [28,29]. They might be born into farm animal groups or habituated to living with farm animals at an early age [9,29]. Guard dogs may stay with the same livestock species throughout their lives, but can also change protection tasks, locations and protected animal species [27]. Guard dogs do not establish territories, but rather stay with and protect the livestock groups even when they are moving [9]. Maintaining proximity to the animals is crucial for effective protection, as the dogs should protect the farm animals rather than the territory and move with the animals as necessary [9]. In Switzerland, guard dogs have been shown to be characteristically reliable, attentive and watchful, resulting in an ability to form social bonds with their own and with other species [26].

Learning about the specific threats facing each protected species and the normal situation of each grazing site requires cognitive effort by the dogs. They must distinguish between a threat to the livestock and the presence of, for example, hikers, external dogs and mountain bikers, and not all potential guard dogs are able to do this [27,30]. Although guard dogs are bred to work independently and without human guidance, they still need to be socialised to humans and the specific conditions of each farm [26]. Some guard dogs have only occasional contact with people, while others live in close proximity, for example, in Switzerland, the dogs guarding sheep may even live in the shepherd’s house [26]. Guard dogs not socialised to humans may be difficult to handle and to treat when needed [26], and public debate has arisen over whether it is safe to pass a guarded farm animal group, especially for a child or a domestic dog [27]. It has been suggested that working guard dogs should be at least 2 years of age to be reliable in this respect [10]. There has also been debate over the preferable sex, and whether a guard dog should be intact or neutered, for it to be reliable and not distracted during the dogs’ mating season [10]. In addition, there has been a so far unresolved discussion over the optimum number of guard dogs necessary for groups of most species [25], although three guard dogs per 100 animals has been claimed to be a suitable ratio for sheep [31]. In a nutshell, patience, time and close observation are needed to keep guard dogs and to acclimatise the animal groups to the presence of the dogs [30]. Nevertheless, an increase in guard dog use of 5% has been reported year on year in Australia [24].

In Germany, guard dogs have been imported from Asian, Middle, and Eastern European countries. For example, the Turkish Kangal, the French Great Pyreneen, the Kaukasian Owtscharka, the Sarplaninac from Balkan countries, the Spanish Mastino Español, the Italian Maremmano Abruzzese, and dogs of mixed guard dog breeds are now kept and bred in Germany (see [26] for an overview on guard dog breeds).

Most of the research to date on guard dogs has been conducted in the USA [10], Canada [25], Switzerland [26] and Australia [9,24,27], where farms and farming practices are generally very different from one country to another. To assess guard dog use for protecting a range of farm animals in Germany, we studied 113 guard dogs on German farms using two approaches: I) we used direct field observation to assess guard dog proximity to, and behaviours towards goats and horses. II) We asked equine science and agriculture students trained in behaviour observations, and official herd management commissioners, to report their experiences with guard dogs during their initial visits to farms keeping various livestock species. These reports, provided through recollection protocols, included observations of the dogs’ behaviour, as well as information about farm and dog management practices. We asked: a) How long have the guard dogs been kept at the farm and how many do they have? b) Which animal species are protected and what are the tasks of the guard dogs? c) Where on the farm are the guard dogs kept and how are they socialised? d) Which ages, sex, and breeds of guard dogs are kept? e) How do guard dogs behave towards their own species, various farm animal species, their owner, and towards known and unknown external persons or dogs?

## Methods

### Research period and regions

**Fig 1:**
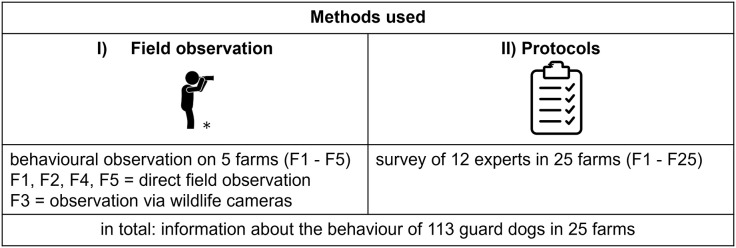
Observation methods. See details at Table 1 and S1 Table. *copyright free figure by Vecteezy.com.

Data for the present study were collected from 25 farms between 1. May 2022 and 31. January 2024 by two methods: I) field observation and II) recollection protocols (Fig 1). We received recollection protocols from visiting herd protection experts from all 25 farms, but the owners of only five farms gave us permission to observe guard dogs and the guarded animals by direct field observations (Table 1).

**Table 1:** Information on the farms and their livestock, and on the applied observation method.

farm	guard area in m²	guard dog numbers, breeds	livestock at farms	guarding task	change of tasks	location guard dogs	species sampled/ sampling method
F1	2000	2, Kangal	20 sheep, 40 horses	livestock groups, the farm	no	on farm, in the house	horses - observation / all – protocol
F2	1000	2, Great Pyreneen	5 horses	the farm	no	fenced area with livestock, on farm at fenced area	horses - observation / all – protocol
F3	2000	5, Sarplaninac	65-75 goats, 7 horses, 2 donkeys	livestock groups, the farm	rarely	fenced area with livestock, on farm at fenced area	goats – camera / all – protocol
F4	2228 and 6444	7, Great Pyreneen	NA sheep, NA goats, 2 horses, NA cattle, NA poultry	livestock groups, the farm, the house	frequently	fenced area with livestock, on farm, on farm at fenced area, in the house	horses - observation / all – protocol
F5	16000	6, Kangal, Owtscharka	20 horses, 1 donkey, NA cattle, NA pigs	livestock groups, the farm	frequently	on farm, fenced area	horses - observation / all - protocol
F6	NA	4, Sarplaninac	3 horses	livestock groups	no	fenced area with livestock, on farm at fenced area	all – protocol
F7	NA	6, Kangal, Owtscharka	1600 sheep, 100 goats	livestock groups	no	fenced area with livestock, on farm at fenced area	all – protocol
F8	NA	2, Great Pyreneen	6000 poultry	livestock groups	no	fenced area with livestock	all – protocol
F9	NA	7, Great Pyreneen	150 sheep	livestock groups	no	fenced area with livestock	all – protocol
F10	NA	4, Kangal	50 sheep, 20 goats	livestock groups	no	fenced area with livestock	all – protocol
F11	NA	6, Great Pyreneen	50 cattle	livestock groups	no	fenced area with livestock	all – protocol
F12	NA	4, Mastino Español	150 sheep, 10 goats	livestock groups	no	fenced area with livestock	all – protocol
F13	NA	7, Great Pyreneen	800 sheep, 20 goats	livestock groups	no	fenced area with livestock	all – protocol
F14	NA	6, Owtscharka	400 sheep, 100 goats	livestock groups, the farm	no	fenced area with livestock, on farm at fenced area	all – protocol
F15	NA	2, Maremmano Abruzzese	400 sheep	livestock groups	no	fenced area with livestock	all – protocol
F16	NA	4, Great Pyreneen	5 sheep, 16 horses, 30 cattle	livestock groups	no	fenced area with livestock	all – protocol
F17	NA	7, Kangal, Owtscharka, Mastino Español, Mix	600 sheep, 150 goats, 10 horses, 1 donkey, NA cattle	livestock groups	rarely	fenced area with livestock, on farm at fenced area	all – protocol
F18	NA	4, Great Pyreneen	950 sheep	livestock groups	no	fenced area with livestock	all – protocol
F19	NA	2, Great Pyreneen	800 poultry	livestock groups	no	fenced area with livestock	all – protocol
F20	NA	2, Great Pyreneen	250 sheep	livestock groups	no	fenced area with livestock	all – protocol
F21	NA	4, Mix	3 horses	livestock groups, the farm	no	fenced area with livestock, on farm at fenced area	all – protocol
F22	NA	5, Great Pyreneen	250 sheep	livestock groups	NA	fenced area with livestock	all – protocol
F23	NA	6, Kangal, Great Pyreneen	2350 sheep, 150 goats, 4 horses, 4 donkeys	livestock groups, the farm	frequently	fenced area with livestock, on farm, on farm at fenced area	all – protocol
F24	NA	5, Sarplaninac	1300 sheep, 6 donkeys 1200 poultry	livestock groups	no	fenced area with livestock, on farm at fenced area	all – protocol
F25	NA	4, Great Pyreneen	NA cattle	livestock groups	no	fenced area with livestock	all – protocol

Missing data are indicated by NA. For more detail see S1 and S2 Tables.

Between 1. May 2022 and 31. October 2023, we observed 19 guard dogs (Fig 1) when guarding farm animals at five farms (F1 – F5; Fig 1; Table 1; S1 Table) in the German states of Bavaria, Baden-Württemberg, Lower Saxony and Rhineland-Palatinate. The median size of the observed areas was 2,000 m² (min. = 500 m², max. = 6,444 m²). On three farms the guard dogs changed pastures with the animals they guarded, and on the remaining two farms they stayed on permanent pastures. On four farms (F1, F2, F4, F5; Fig 1; Table 1) the experimenters sampled the data of 15 guard dogs when guarding horses in direct, continuous, all occurrence observations and at a fifth farm (F3; Fig 1; Table 1) we placed 2 wildlife cameras, type Diftal Trail by Coolife, in two enclosures to photo-sample 4 guard dogs and guarded goats continuously.

All guard dogs and farm animals were kept in accordance with their individual needs. The animals were in a median body condition score of 3 (min. = 2, max. = 4) on a scale of 1 for emaciated and 5 for obese. They had permanent access to water, were fed individually with species specific feed, were checked daily for welfare and feeding status, and received regular veterinary care.

Between 1. October 2023 and 31. January 2024, twelve experts, trained in observing guard dogs, reported via a recollection protocol (Fig 1; S2 Table; S1 File) their contact over the preceding year with 113 guard dogs guarding various farm animal species. These protocols pertained to their first visits to 25 farms (F1 – F25; Fig 1; Table 1) in the German states of Bavaria, Baden-Württemberg, Saxony, Lower Saxony, Rhineland-Palatinate, North Rhine-Westphalia, Hessen, Hamburg, Mecklenburg-West Pomerania and Schleswig Holstein.

### Ethical considerations

This study is registered under the numbers: 2022_32_10.04.2022; 2022_64_17.05.2022; 2023_61_11.03.2023 at Nuertingen-Geislingen University. Permission was given from the Ethics Board of Nürtingen-Geislingen University, as the claims of the Data Protection Directive of the European Union (DSGVO 2016) were fulfilled. The observation protocols, the video material, showing the experimental procedure, and the recollection protocols of the experts were transferred into anonymous, written form directly after conducting the material. From the anonymous raw data, no individual data could be assigned to particular persons. Only the written, anonymous raw data was used for further analysis for the study. The methods of the study were visualized in graphs without showing persons. The study published no pictures, videos or any data from which personal data could be drawn, either in the manuscript or in any supplementary material. The participating persons took part in the study between 1. May 2022 and 31. January 2024 and declared written consent a) for participating in the data curation, b) for documenting their personal data on the recollection protocols, c) for transferring the data into written form, and d) for using the anonymous, written raw data for the present publication. The informed consent can be viewed on request.

### Animals

#### Guarded farm animals.

A number of different farm animal species were protected by guard dogs (Table 1): they protected sheep at 15 farms, goats at 8 farms, cattle at 6 farms, horses at 9 farms and poultry at 4 farms. Four farms kept donkeys and 1 farm kept pigs. The farms kept a median of 2 animal species (min. = 1 species, max. = 6 species; which could be sheep, goats, cows, horses, poultry, pigs, donkeys) and a median of 161 farm animals (min. = 3 animals, max = 6,000 animals). The livestock were kept in groups on pasture, either permanently or during the day. The ground in shelters and feeding areas was covered with straw or wood shavings.

#### Guard dogs at the farms.

In total we analysed the behaviour of 113 guard dogs. We observed 19 dogs at five farms with field observations (field observation: F1 - F5; Table 1; S1 Table; note: the five farms kept 22 guard dogs, but only 19 were included in the observations) and received behaviour protocols for all 113 guard dogs across all 25 farms (protocols: F1 – F25; Table 1; S2 Table; S1 File). The protocols were compiled from the initial visits by the experts to the farms. The experts comprised 13 equine science and agriculture students trained in behaviour observations, and 6 official herd management commissioners. The guard dogs were kept in groups of a median of 4 animals (min. = 2, max. = 7) and roamed freely in fenced areas with permanent shelter and sleeping areas at their farms.

The tasks of the guard dogs differed between the farms (Table 1): 1 farm kept dogs to guard the farm, 17 to guard livestock herds, 6 to guard the farm and the livestock, and 2 farms kept dogs to guard the farm, the livestock and the house. The tasks of the guard dogs were constant at 19 farms and changed at 5 farms, and at one farm, this information was not available. 21 farms bought guard dogs specifically for protection against wolves. Each guard dog group protected a median of 1 animal group (min. = 1 group, max. = 5 groups).

The farms had kept guard dogs for various lengths of time: less than 1 year (N = 2), 1–5 years (N = 8), 6–19 years (N = 11) and more than 10 years (N = 2). The guard dogs were socialized to persons and farm animals to five levels, with 1 representing the highest and 5 the lowest degree of socialization:

1 = Guard dogs arrived at the farm as puppies and were then socialised to other animals and humans (N = 40).

2 = Guard dogs arrived as puppies and were then socialised only to other animals (N = 41).

3 = Guard dogs arrived as mature animals and were socialised to other animals and humans thereafter (N = 9).

4 = Guard dogs arrived as mature animals and were socialised to other animals thereafter (N = 4).

5 = Guard dogs arrived as mature animals and were socialised to humans thereafter (N = 19).

We categorised the farms according to the age groups of the guard dogs kept: young (aged 1–2 years) at 7 farms, mature (aged 2–10 years) at 11 farms, old (aged more than 10 years) at 1 farm, and all ages (from less than 6 months to more than 10 years) at 6 farms. At two farms the guard dog groups consisted of only males, at three farms of only females, and at 20 farms of males and females. One farm kept only neutered guard dogs, twelve kept only intact guard dogs and twelve a mix of neutered and intact guard dogs. The guard dogs were of various breeds (Kangal, Great Pyreneen, Kaukasian Owtscharka, Sarplaninac, Mastino Español, Maremmano Abruzzese, and dogs of mixed guard dog breeds; Table 1). All dog data are provided in the S1 and S2 Tables.

### Experimental procedure

I) **Field observation**

At five farms we observed the guard dogs’ behaviour towards and proximity to goats and horses in the field, with field observation methods (Fig 1; Table 1; S1 Table):

a) At four farms (F1, F2, F4, F5; Fig 1), the proximity of 15 guard dogs to horses was recorded every 10 minutes (see description of proximity observations below). Directed behaviour (see description of behaviours below) of the 15 guard dogs was collected continuously, by direct, all occurrence sampling [32] on 3 consecutive days within one week, for a total duration of 10.5 hours (median, min. = 7 h, max. = 22 h). When a behaviour display continued for more than 10 seconds (for example, barking) it was counted again for the particular animal. The four farms kept horses and other farm animals (Table 1), and were visited by 3 to 6 experimenters. The experimenters met the owners for an introduction to the farm before they started documenting the guard dogs’ and horses’ proximity and behaviour.

**Habituation phase:** Before starting the recordings, the animals were habituated to the presence of the observers who stood at 2 or 3 different positions in the animals’ enclosures, at a distance of 50 meters from the animals, for 1 hour, while the identity of the animals was noted on paper and distributed between the observers. When the observed animals ignored the experimenters, the behaviour recording was started.

b) At farm F3 (Fig 1) we placed two wildlife cameras, type Diftal Trail by Coolife, in two enclosures to take continuous picture sampling of guard dogs and the two groups of guarded goats (Table 1). The proximity and undirected behaviour of particular guard dogs and the nearest goat (the focus goat) were documented from selected pictures (selection procedure see Data processing). Group 1 had two guard dogs, and was recorded for 123 days, group 2 had two other guard dogs and was recorded for 102 days. Farm F3 had 6 guard dogs in total, of which 4 constantly stayed with the goats. The cameras were placed within each enclosure at the area most frequently visited by the animals, and in such a way that they would only record the area inside the enclosure. When a particular group moved to a new enclosure, the cameras were moved to the new location. The cameras were checked daily, pictures showing persons were deleted and the remaining pictures were stored at a laptop.

**Habituation phase:** For proximity and behaviour recordings with the camera, no habituation phase was needed. The identity of the guard dogs was clearly visible on the camera recordings. The goats’ identities were not recorded. For any picture, we recorded an unidentified, nearest goat to an identified guard dog for proximity analysis, and the behaviour of that particular dog.

II) **Protocols**

19 experts, comprising 13 equine science and agriculture students trained in behaviour observations and 6 official herd management commissioners, reported their experiences with 113 guard dogs, which were guarding various farm animal species, on their first visits to the farms in the preceding year with recollection protocols (Fig 1; Table 1; S2 Table) and returned the protocols on paper (S1 File) via email. For five farms (F1 – F5) we received multiple recollection protocols (median = 3, min. = 2, max. = 4) which were considered to be reliable, as inter-observer ratings revealed significant conformity between the protocols (Spearman rank correlation test: all p < 0.05; full test results see S2 File).

All experts were asked to describe the guard dogs and farm animals at a particular farm, the farmers’ experience in guard dog management, the tasks of the guard dogs, the guard dogs’ prior socialization, and the guard dogs’ behaviour (for detail see further below). Most questions were closed, only the last question was open and invited the experts to report any outstanding experiences at the respective farm [33].

#### Experimenters.

I) **Field observation**

Between 1. May 2022 and 31. October 2023, between three and six experimenters participated in each observation. They were young women (between 20 and 25 years old), trained in animal behaviour observations, and comprised one biology, and 30 agriculture and equine science students. They shared experimental tasks, such as:

- Staying passive within the enclosure while waiting for the animals to habituate to the recordings
- Animal identification
- Distributing themselves over the experimental enclosure and roaming with the animals
- Documenting proximity and behaviours in the field, on paper or from camera pictures, on a laptop

II) **Protocols**

Between 1. October 2023 and 31. January 2024, 19 experts, of whom 12 also participated in the field observations, were contacted via telephone or email and completed a protocol reporting their experiences with guard dogs during their first visits to farms in the preceding year.

### Proximity between guard dogs and guarded farm animals

From the data of the field observations (farms F1 - F5; Fig 1; Table 1; S1 Table), we recorded the proximity between the guard dogs and goats and horses. In direct observation (farms F1, F2, F4, F5) the experimenters drew a distribution graph of the observed animals every 10 minutes and recorded the distance between all the animals on paper. The data on the proximity frequencies for the particular guard dogs and each guarded farm animal was then transferred to an excel sheet. When recording the animals with cameras (farm F3), the distance between the particular guard dog and the nearest goat (the focus goat) was documented from each picture and the proximity frequencies were noted on excel.

We recorded the following proximity categories [34]:

- Contact = guard dogs and farm animals were in direct, physical body contact
- 1 body length = guard dogs and farm animals were within one farm animal body length
- 1–3 body lengths = guard dogs and farm animals were within one to three farm animal body lengths.
- > than 3 body lengths = guard dogs and farm animals were more than three farm animal body lengths apart.

### Behaviour observations

The behaviour of the guard dogs was recorded for both data collection methods, as defined by [35,36]. We summed up the dogs’ behaviours in the below mentioned categories:

1. **Friendly – attentive**: sitting, looking in direction of someone, pricking up ears, sniffing, wagging the tail, friendly approach, feeding together, grooming, howling, playing, following.
2. **Neutral – relaxed:** lying, no response, sleeping, yawning, stretching, scratching, rolling, feeding alone, drinking alone.
3. **Anxious:** standing with tail down, keeping distance, hiding head and tails, running back and forth, retreating.
4. **Dominant – aggressive:** standing with tail up, dominant posture, mounting, passive and active threatening, barking, biting, chasing, growling, attacking, focusing, baring teeth.

We recorded the recipient of guard dog behaviours from direct field observation (I; F1 – F5; Table 1; S1 Table) and the protocol data (II; F1 – F25; Table 1; S2 Table).

I) **Direct field observation data revealed 19 guard dogs’ behaviour towards**:
  - Own species: other members of the guard dogs’ group
  - Guarded species: the goats or horses the guard dogs were kept with
  - Others: individuals outside the fenced areas (we recorded: persons, dogs, cats, deer, birds, bikers, cars)
II) **Using the protocols, experts reported their recollections of all 113 guard dogs’ directed behaviours towards specific individuals, including:**
  - Owner: has owned and taken care of the guard dog since its birth, or for at least 6 months
  - Farm animals: all animal species living at the particular farm
  - External dogs: dogs not part of the guard dog group, outside or inside the guard dogs’ territory
  - Known person in the presence of the owner: guard dog was in contact with the known person at least once before, owner visible, audible or sniffable to the guard dog.
  - Unknown person in the presence of the owner: guard dog never in contact with the unknown person before, owner visible, audible or sniffable to the guard dog.
  - Known person in the absence of the owner: guard dog was in contact with the known person at least once before, owner not visible, audible or sniffable to the guard dog.
  - Unknown person in the absence of the owner: guard dog never in contact with the unknown person before, owner not visible, audible or sniffable to the guard dog.

### Data

#### Data processing.

The camera recordings at farm F3 (I, b; Table 1) resulted in 25,000 pictures. Pictures with a person or pictures with just the guard dogs without goats or just the goats without guard dogs were excluded. This resulted in 3,200 pictures from the total observation period. From these, one picture per hour was randomly selected, resulting in 513 images for group 1 with two guard dogs, and 239 images for group 2 with another two guard dogs.

The data from the camera recordings, the direct field observations, and the recollection protocols were transferred to excel sheets. To achieve population level comparability between the protocols and the field observations, the data had to be adjusted:

- When data from several categories were mentioned, we chose the category which was most frequently reported for each particular observation or report.
- When undirected behaviours or proximities were recorded, we summed behaviours of each category and proximities of each category.
- The behaviour observation and proximity counts were adjusted to unequal durations of observations as follows:
  - behaviour observation counts / hours of observation or number of pictures = behaviours per hour.
  - proximity counts / hours of observation or number of pictures = proximity counts per hour.

#### Data analysis.

The software of the R statistical environment (R Development Core Team, Boston MA, USA, version 4.4.2) with its package Rstudio and Rcommander was applied for data analysis and data visualization, and Excel 2016 for listing and organizing the data. Most data were not normally distributed (Shapiro-wilks test: most p < 0.05), therefore we applied non-parametric tests throughout.

We considered the dogs’ behaviour towards guarded animals, own species, persons and others, and their proximity to guarded animals, as main components and analysed whether their mutual eigenvectors would overlap in three components with Principal Component Analyses (PCAs). The PCA revealed equal proportions of variance for the main directed behaviour components of the protocols and the proximity measures in the first eigenvector, but the directed behaviour of the field observation overlapped in the first and second eigenvector (S3 File). Therefore, we continued by comparing the main components (dogs’ behaviour towards guarded animals, own species, persons and others and their proximity to guarded animals) of the protocols and the field observation with each other using non-parametric variance analyses (Friedman rank sum tests and Wilcoxon signed rank tests; S3 File).

Thereafter, we analysed the impact of the factors by applying separate Generalized Linear Models (GLMs) for each main component (S3 File). For the data of the field observation, we used the following formulas to analyse effects on the proximity and the behaviour categories of the guard dogs: glm (formula = proximity ~ age status + breed + farm + sex intact + number of animals guarded + observation direct or camera + sex + size of guard dog group + size of guarded area + socialization, family = gaussian(identity), data = Dataset) and glm (formula = behaviour category ~ age status + breed + farm + sex intact + number of animals guarded + observation direct or camera + sex + size of guard dog group + size of guarded area + socialization, family = gaussian(identity), data = Dataset). For the data of the protocols, we used the formulas: glm (formula = target of behaviour ~ age group + breeds + sex intact + location at farm + observer + sex + socialization + number of animals at farm + duration owner has guard dogs + bought for wolf protection + groups of animals protected + number of guard dogs + number of species at farm + task change + tasks, family = poisson(identity), data = Dataset. To reduce the number of factors for each model, we analysed a potential mutual overlap of factors in three eigenvectors with a factor analysis and conducted one GLM for a main component when all its factors overlapped in the first eigenvector and two GLMs when the factors overlapped in two eigenvectors. Then, we reduced the factors of each GLM by choosing the model with the lowest Akaike Information Criterion (AIC [37]). All factors under debate were fixed factors, set by the experiment.

The significance level was set at 0.05 for all tests, the confidence interval was 95%. When data were used for multiple testing with conventional test procedures, we corrected the significance level by applying a sequential Bonferroni correction [38] and only the p-values which were smaller than the corrected significance level were defined ‘significant’. All tests were two-tailed. Please see the complete statistical data at the supporting information S3 File.

## Results

Detailed information on the length of time the guard dogs had been working on particular German farms, the location where the guard dogs were kept on the farms, the tasks of the guard dogs and which animal species they protected, how the guard dogs were socialised, the guard dogs’ age, sex, whether they were neutered or intact, and their breed, are provided in the Method section, Table 1, and supporting information S1 and S2 Tables. These data were considered factors potentially affecting the proximity guard dogs maintained to the livestock and the behaviour of the guard dogs towards their own species, external individuals, the guarded species, their owner, and towards known and unknown external persons and dogs. The results are provided below.

I) **Field observation**
**Observed proximity between individual guard dogs, and goats and horses (I; F1 – F5)**

**Fig 2:**
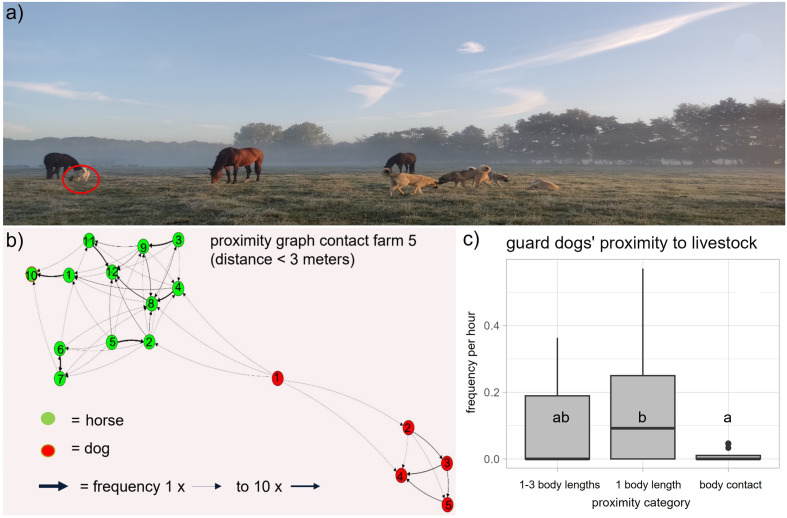
The observed guard dogs’ proximity to goats and horses. a) Snapshot from observations at farm 5: while the female dog (far left, red circle) stayed in close contact with a horse, her offspring kept a distance of 3 horse body lengths to the closest horse. Reprinted under a CC BY 4.0 license, with permission from Sarah Handel, original copyright 2025. b) Proximity graph of the frequencies the dogs (red) stayed in close contact with horses (green) at farm 5: while the female dog (dog1) was observed in close contact with three horses once, her offspring (dogs 2–5) were never seen in close contact with any horse. c) Proximity categories of dogs to livestock at farm 1–5, in frequencies per hour: the proximity was categorised in 1-3 body lengths, in 1 body length, and in contact with a farm animal. The dogs stayed less in direct contact with the farm animals (a) than within one body length (b) (Wilcoxon signed rank tests: *N* = 19, *V* = 0, *p* = 0.009).

The guard dogs (N = 19) were observed to be in body contact with goats and horses with a median frequency of 0 per hour (min. = 0 / h, max. = 0.05 / h), in the proximity of 1 body length of the guarded animal with a median frequency of 0.19 per hour (min. = 0 / h, max. = 0.57 / h), and in the proximity of 1–3 body lengths of the guarded animal with a frequency of 0 per hour (min. = 0 / h, max. 0.36 / h). Body contact between guard dogs and goats and horses was observed significantly less frequently than staying within a proximity to farm animals of 1 body length (Wilcoxon signed rank tests: *N* = 19, *V* = 0, *p* = 0.009; Fig 2; S1 Table)

**Factors on the frequency of observed proximity between individual guard dogs and goats and horses (I; F1 – F5)**

**Fig 3:**
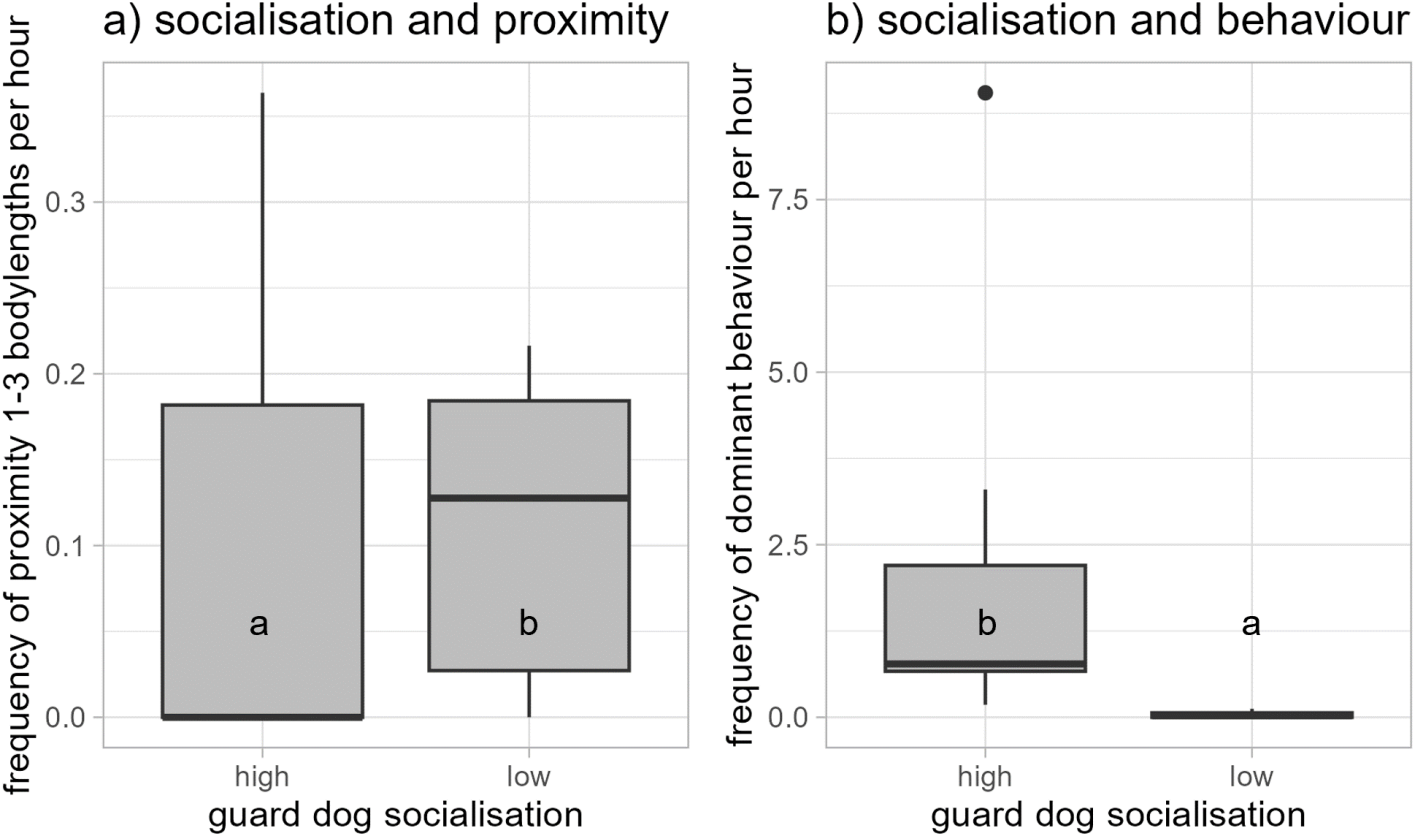
The effect of the guard dogs’ socialization on their proximity of 1–3 body lengths with goats and horses and their behaviour towards their own species, farm animals, farm persons and others. a) guard dogs on a high degree of socialization (degree 1 = guard dogs arrived at the farm as puppies and were then socialised with other animals and humans) were seen less in the proximity of 1–3 body lengths to goats and horses than those on a lower degree (degree 3 = guard dogs arrived as mature animals and were socialised with other animals and humans thereafter) and **b)** guard dogs with high degree of socialization displayed more dominant behaviour than those with a lower degree of socialization.

The guard dogs preferentially stayed within 1 body length of the guarded animals (Fig 2). Additionally, a proximity of 1–3 body lengths was observed less frequently when they guarded goats, which were recorded with cameras (median = 0.17 / h, min. = 0.11, max. = 0.22) than when guarding horses, which were recorded in direct field observation (median = 0, min. = 0, max. = 0.36) (GLM: *N* = 19, *t* < −0.001, *p* < 0.001). Furthermore, guard dogs were seen less frequently in the proximity of 1−3 body length to goats and horses the more animals they guarded (GLM: *N *= 19, *t* < −0.001, *p* = 0.001; Table 1), and less frequently *t*he better they were socialised to farm animals and persons (GLM: *N* = 19, *t* < −0.001, *p* < 0.001; Fig 3).

The frequency with which guard dogs stayed in the close proximity of goats and horses was affected by the size of the guard dog groups, i.e., the larger the guard dog group the less frequently they stayed in direct contact (GLM: *N* = 19, *t* = −2.422, *p* = 0.03), but the more frequently they stayed within 1 body length (GLM: *N *= 19, *t* = 4358, *p* < 0.001) and 1–3 body lengths of the livestock (GLM: *N* = 19, *t* < 0.001, *p* < 0,001).

**Fig 4:**
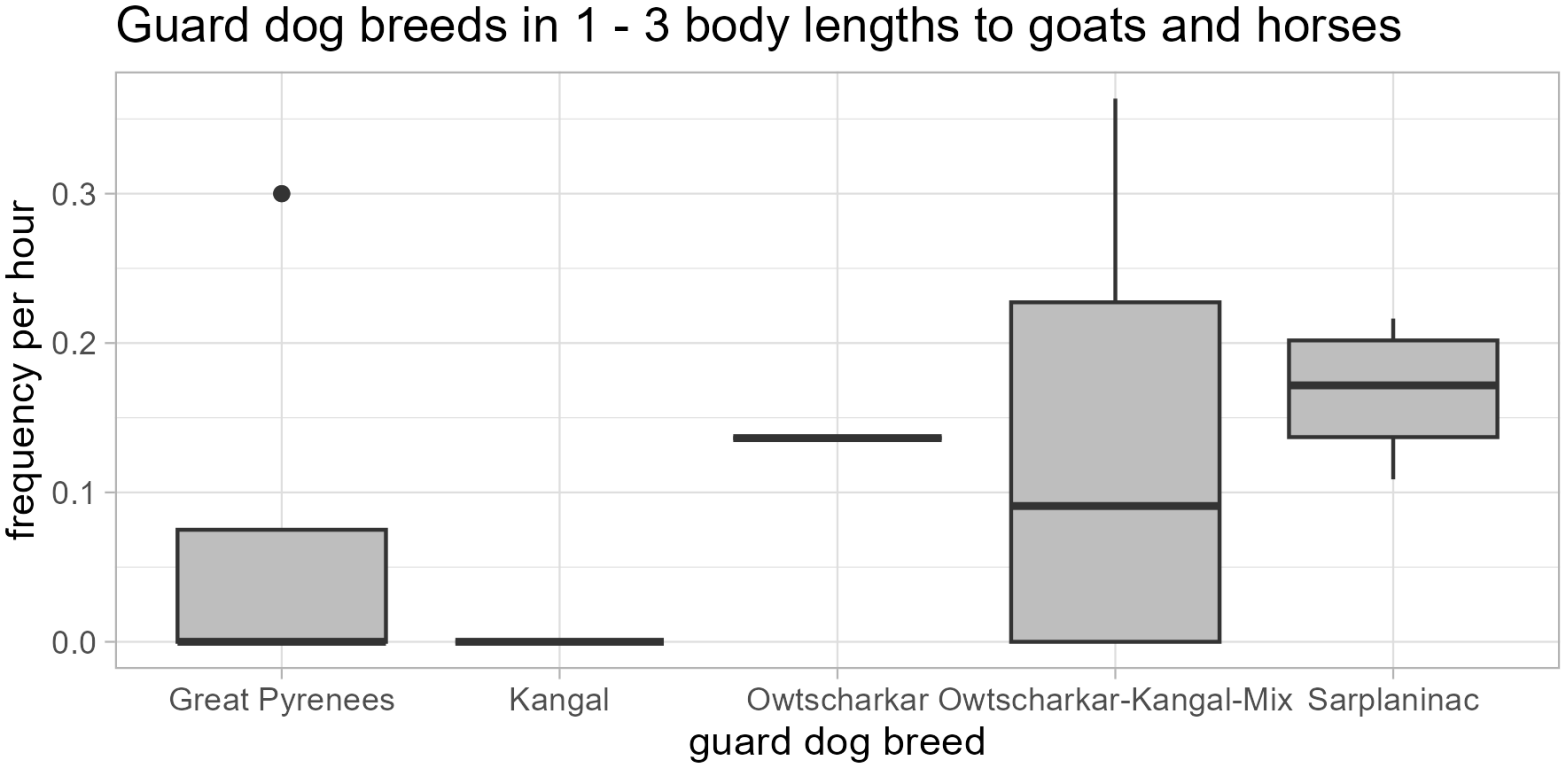
Guard dogs (N = 19) by breed, in proximity of 1–3 body lengths to goats and horses. The frequency with which guard dogs stayed within 1–3 goat and horse body lengths to the protected animals varied according to their breed (GLM: *N* = 19, *t* = −3.26, *p* = 0.01), with Great Pyrenees (N = 8) and Kangals (N = 2) less frequently and Owtscharkar (N = 1), Owtscharkar-Kangal Mix (N = 4) and Sarplaninac (N = 4) guard dogs more frequently (F1 – F5; Table 1).

The frequency with which they stayed within the proximity of 1–3 body lengths to goats and horses also differed between breeds (GLM: *N* = 19, *t* < 0.001, *p* < 0.001; Fig 4). Great Pyrenees (N = 8) were within 1–3 body lengths to the guarded animals with a median frequency of 0 per hour (min. = 0 / h, max. = 0.3 / h), Kangals (N = 2) with a frequency of 0 times per hour, Sarplaninacs (N = 4) with a median frequency of 0.17 per hour (min. = 0.11, max. = 0.22), Owtscharkars (N = 1) with a frequency of 0.14 per hour, and Kangal-Owtscharkar cross breeds (N = 4) with a median frequency of 0.09 per hour (min. = 0, max. = 0.36; Fig 4).

However, whether guard dogs stayed in close body contact or within 1 body length of goats and horses was not affected by the guard dogs age, breed, sex, whether they were intact or neutered, the farms they were kept at, the guard dogs’ socialization, the size of the guarded area, the number of animals they guarded, or whether they were observed directly or with a camera (GLM: *N* = 19, all *p* > 0.05). See statistical results for all factors at S3 File.

**Observed individual guard dogs’ behaviour towards farm animals, farm persons and others (I; F1 – F5)**

**Fig 5:**
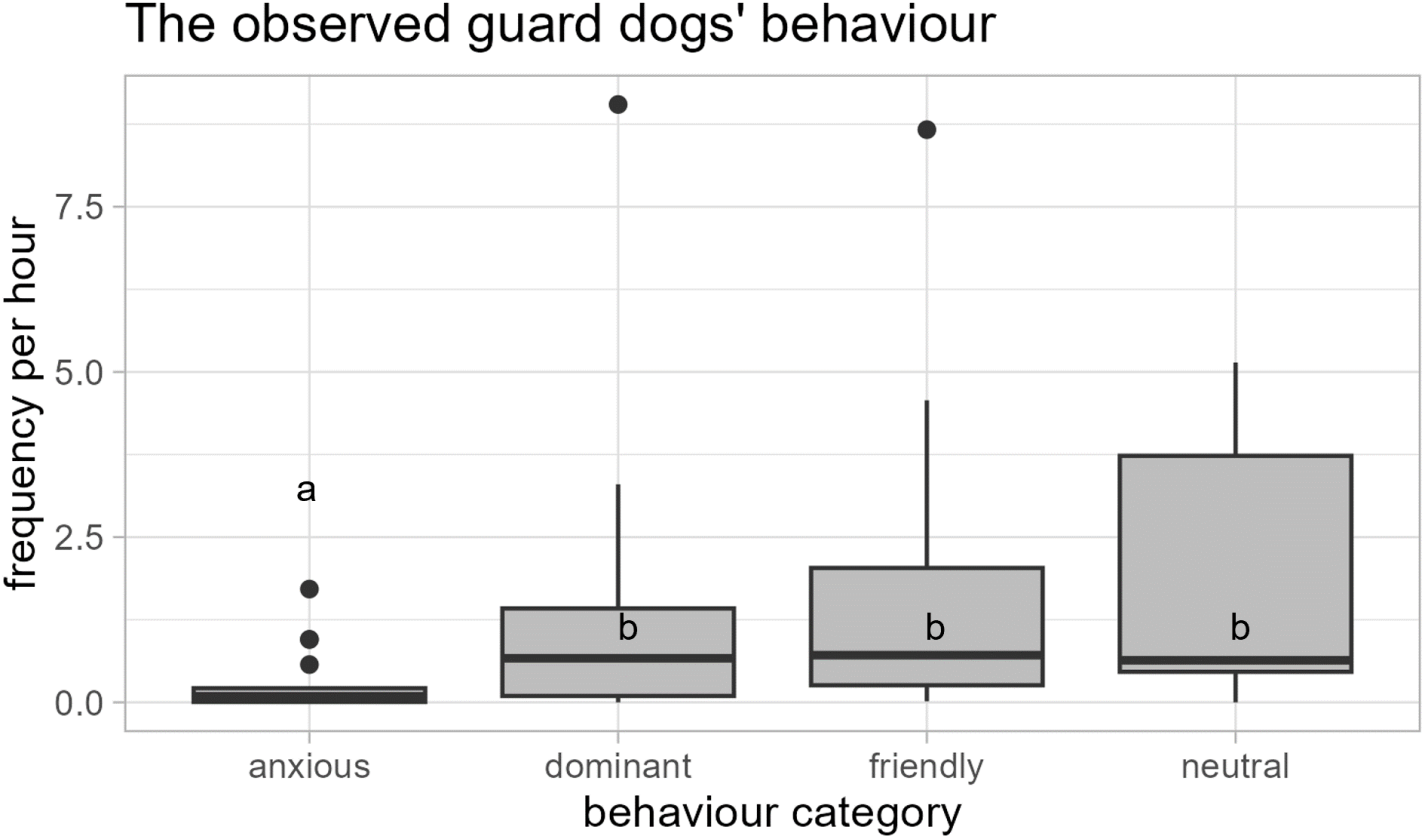
The observed guard dogs’ behaviour towards their own species, farm animals, farm persons and others. The behaviour of 19 guard dogs was observed when they were guarding goats and horses at their farms and was categorized as 1) friendly, 2) neutral, 3) anxious, and 4) dominant. The dogs showed significant less anxious behaviour (a) than behaviour from any other category (b; Wilcoxon signed rank tests: *N* = 19, all *p* < 0.004).

When guarding goats and horses, guard dogs (N = 19) were observed showing friendly-attentive behaviour with a median frequency of 1.1 per hour (min. = 0.02 / h, max. = 8.67 / h), neutral-relaxed behaviour with a median frequency of 1 per hour (min. = 0 / h, max. = 5.14 / h), anxious behaviour with a median frequency of 0.095 per hour (min. = 0 / h, max. = 1.71 / h), and dominant-aggressive behaviour with a median frequency of 0.76 per hour (min. = 0 / h, max. = 9.05 / h; Fig 5) towards their own species, farm animals, farm persons and others (external persons, dogs, cats, deer, birds, bikers, cars).

They showed significantly less anxious behaviour than any other behaviour category (Wilcoxon signed rank tests: N = 19: anxious versus friendly, *V* = 3, *p* < 0.001; anxious versus neutral, *V* = 0, *p* = 0.001; anxious versus dominant, *V* = 3, *p* = 0.003; Fig 5).

**Factors affecting the frequency of observed individual dogs’ friendly, neutral, anxious and dominant behaviour towards their own species, farm animals, farm persons and others (I; F1 – F5)**

We found more neutral behaviour towards their own species, farm animals, farm persons and others (external persons, dogs, cats, deer, birds, bikers, cars) in the direct observations when dogs guarded horses (median = 1,75 / h, min. = 0 / h, max. = 5.14 / h) than in the camera recordings when they were guarding goats (median = 0.4 / h, min. = 0.28 / h, max. = 0.51 / h) (GLM: *N* = 19, *t* = 3.555, *p* = 0.003). The frequency with which individual dogs displayed anxious behaviour differed between farms (GLM: *N* = 19, *t* = 3.846, *p* = 0.001) and the frequency of neutral behaviour increased when the number of guarded goats and horses increased (GLM: *N* = 19, *t* = 2.251, *p* = 0.04) and decreased when the guarded area increased (GLM: *N* = 19, *t* = −3.403, *p* = 0.004). Guard dogs with a high degree of socialization (degree 1 = Guard dogs arrived at the farm as puppies and were then socialised with other animals and humans) were more dominant – aggressive than guard dogs with a lower degree of socialization (degree 3 = Guard dogs arrived as mature animals and were socialised with other animals and humans thereafter; GLM: *N *= 19, *t* = −3.515, *p* = 0.003; Fig 3). Note: degrees of socialization other than 1 and 3 were not reported in this context.

Adult animals showed more friendly-attentive behaviour (GLM: *N* = 19, *t* = 2.19, *p* = 0.04) and dominant-aggressive behaviour (GLM: *N* = 19, *t* = 2.901, p = 0.01) than juveniles towards their own species, farm animals, farm persons and others. The larger the guard dog group, the more friendly behaviour was observed in the individual dogs (GLM: *N* = 19, *t* = 4.867, *p* < 0.001). The frequency of neutral-relaxed behaviour differed between breeds (GLM: *N* = 19, *t* = 2.818, *p* = 0.01; Grea*t* Pyreneen median = 4.8 / h, min. = 2.5 / h, max. = 5.1 / h; Kangal median 0.93 / h, min. = 0.81 / h, max. = 1 / h; Sarplaninac median = 0.4 / h, min. = 0.28 / h, max. = 0.51 / h; Owtscharka 0/h; Owtscharka-Kangal-Mix median = 0.56 / h, min. = 0.54 / h, max. = 0.63 / h). Furthermore, the frequency of individual displays of anxious behaviour differed between breeds (GLM: *N* = 19, *t* = −3.939, *p* = 0.001; Grea*t* Pyreneen median = 0.38 / h, min. = 0 / h, max. = 1.7 / h; Kangal 0 / h; Sarplaninac median = 0.03 / h, min. = 0.01 / h, max. = 0.05 / h; Owtscharka 0 / h; Owtscharka-Kangal-Mix median = 0.09 / h, min. = 0 / h, max. = 0.22 / h). See all results for factors in the S3 File.

**Observed direction of the individual guard dogs’ behaviour (I, F1, F2, F4, F5)**

**Fig 6:**
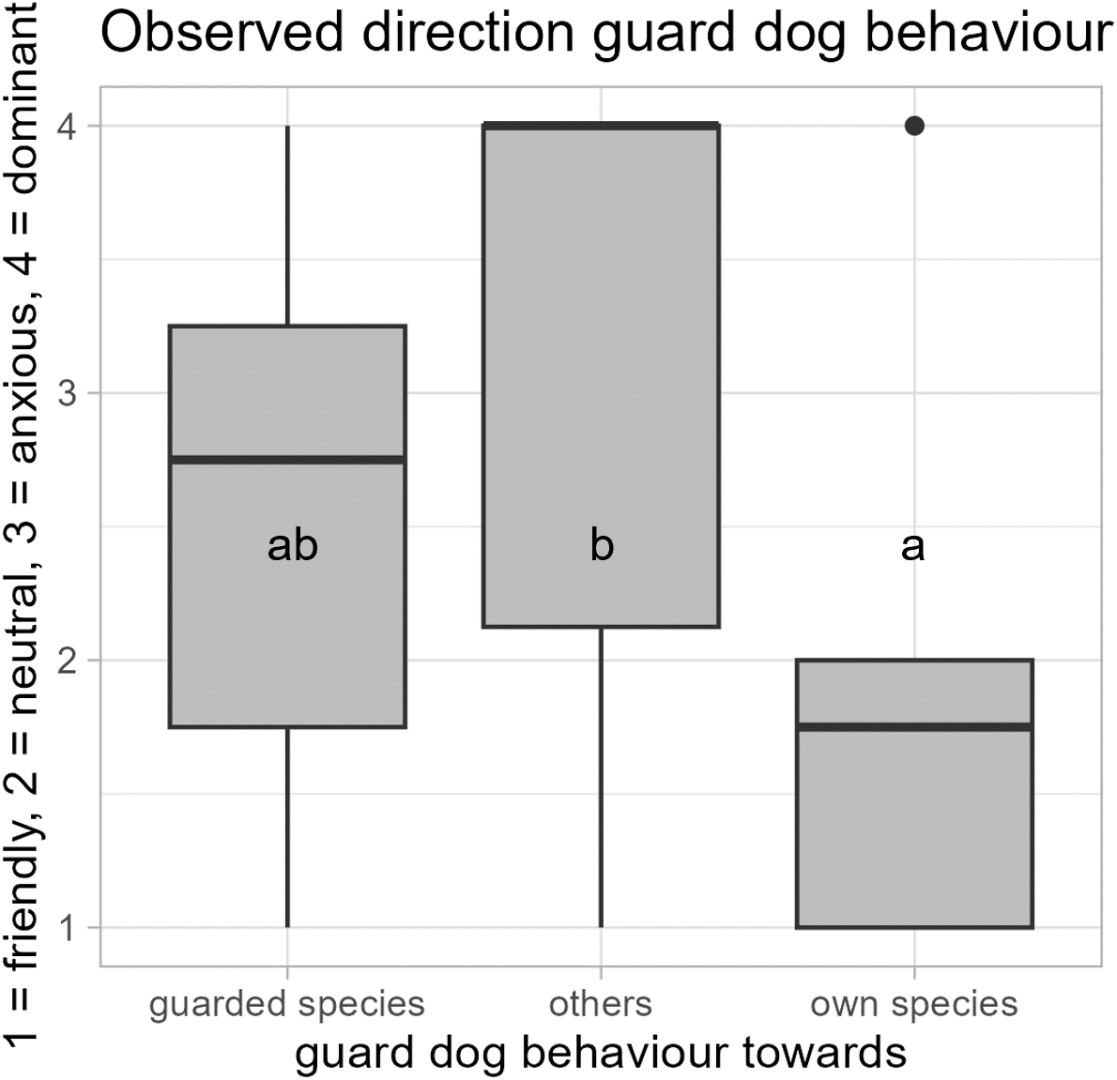
Observed direction of the guard dogs’ (N = 15) behaviour when guarding horses. In direct field observation, the guard dogs’ behaviour varied according to whether it was directed at their own species or others (Wilcoxon signed rank tests: *N* = 15, *V *= 71, *p* = 0.01), and they showed all behaviour categories towards the guarded horses. Guard dogs displayed mostly neutral and friendly and, in one case, dominant behaviour towards their own species, they showed mostly anxious and neutral, but also friendly and dominant behaviour towards the guarded species, and they were mostly dominant, but also anxious, neutral, and sometimes friendly, towards others. The category “others” included external persons, dogs, cats, deer, birds, bikers, and cars.

When the direction of guard dog (N = 15) behaviour was observed when they were guarding horses in direct field observation, they showed a large variety of behaviours, but mostly neutral-relaxed and anxious behaviour towards the horses (median: 2.25, min. = 1 friendly, max. = 4 dominant), mostly dominant-aggressive behaviour towards others (median = 4, min. = 1 friendly, max. = 4 dominant; the category “others” included external persons, dogs, cats, deer, birds, bikers, and cars), and mostly friendly-attentive behaviour towards their own species (median: 1.25, min. = 1 friendly, max. = 2 neutral-relaxed). They were significantly more friendly towards their own species than towards others (Wilcoxon signed rank tests: *N* = 15, *V* = 71, *p* = 0.01; Fig 6).

**Factors affecting the observed direction of the individual guard dogs’ behaviour (I, F1, F3, F4, F5)**

The farms where the guard dogs were kept, the size of the guarded area, the number of horses they guarded, the size of the guard dogs’ group, the guard dogs’ age, sex, whether they were intact or neutered, and their breed did not have any effect on the behaviour category they demonstrated towards the guarded horses, others or their own species (GLM: *N* = 15, all *p* > 0.05; S3 File).

II) **Protocols**
**Reported guard dog behaviour towards their owner, various guarded animal species, external dogs, and external and internal persons in the presence and absence of the owner (II; N = 25 farms)**

**Fig 7:**
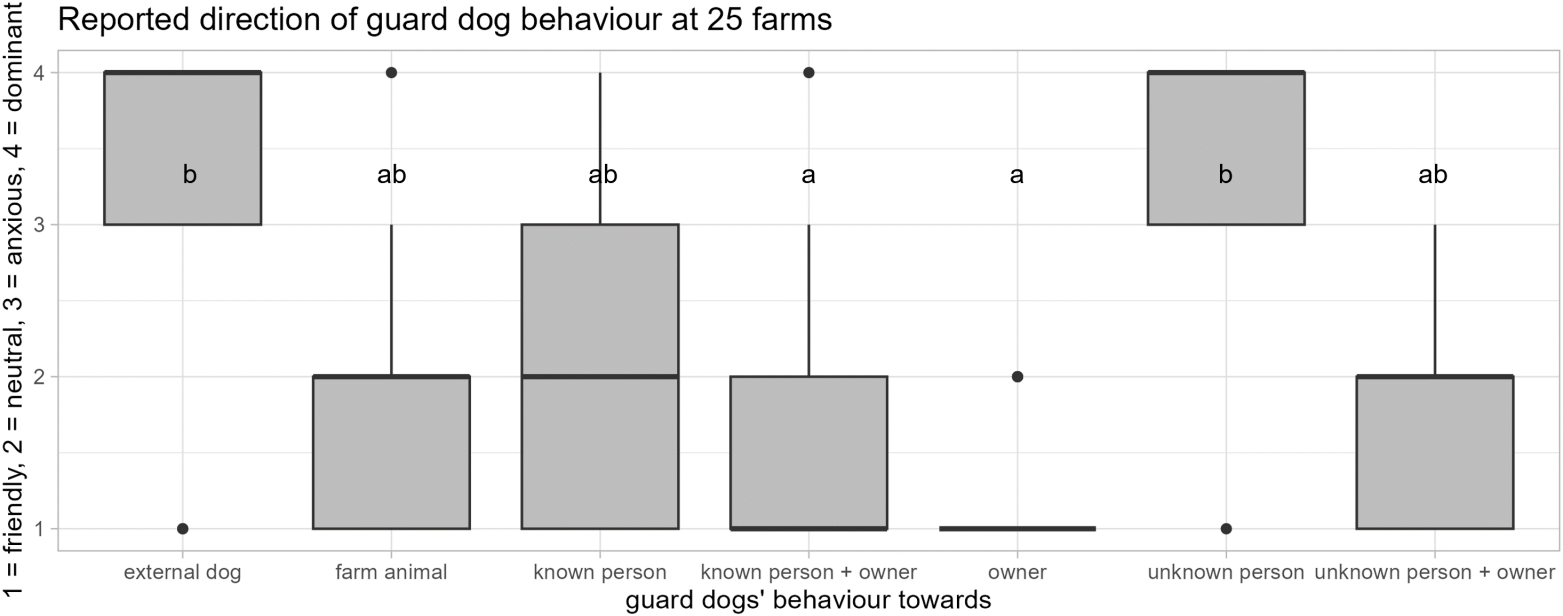
Reported direction of the guard dogs’ behaviour when guarding various farm animal species (N = 25 farms). Based on their first visit to farms with guard dogs, experts reported on the quality of the guard dogs’ behaviour towards various individuals. The dogs showed mostly friendly behaviour towards their owner and towards known persons when the owner was present, while showing mostly dominant behaviour towards external dogs and unknown persons in the absence of the owner (Wilcoxon signed rank tests: *N* = 25, all *p* < 0.05). They displayed a larger variety, but mostly neutral behaviour towards various farm animal species, known persons (ab) and in the presence of the owner also towards unknown persons.

Regarding guard dog behaviour reported from all the farms (N = 25), the dogs guarded a variety of farm animals (Fig 7), and showed exclusively friendly-attentive behaviour towards their owner (median = 1, min. = 1, max. = 2). Furthermore, they showed friendly-attentive and neutral-relaxed behaviour towards farm animals (median = 2, min. = 1, max. = 3), known persons when owner absent (median = 2, min. = 1, max. = 4), known persons when owner present: median = 1, min. = 1, max. = 4) and unknown persons when the owner was present (median = 2, min. = 1, max. = 3). However, they showed anxious and dominant-aggressive behaviour towards other dogs (median = 4, min. = 1, max. = 4) and unknown persons when the owner was not present (median = 4, min. = 1, max. = 4). Guard dogs displayed significantly more friendly-attentive behaviour towards the owner and known persons than towards farm animals in the presence of the owner, towards known persons in the absence of the owner, and towards other dogs and unknown persons with and without the presence of the owner (Wilcoxon signed rank tests: *N* = 25, all *p* < 0.05; Fig 7). Furthermore, guard dogs displayed significantly more dominant-aggressive behaviour towards other dogs and unknown persons in the absence of the owner than towards farm animals, known persons, the owner, and unknown persons in the presence of the owner (Wilcoxon signed rank tests: *N* = 25, all *p* < 0.05; Fig 7).

**Factors influencing reported guard dog behaviour towards their owner, the guarded animal species, external dogs, and external and internal persons in the presence and absence of the owner (II; N = 25 farms)**

**Fig 8:**
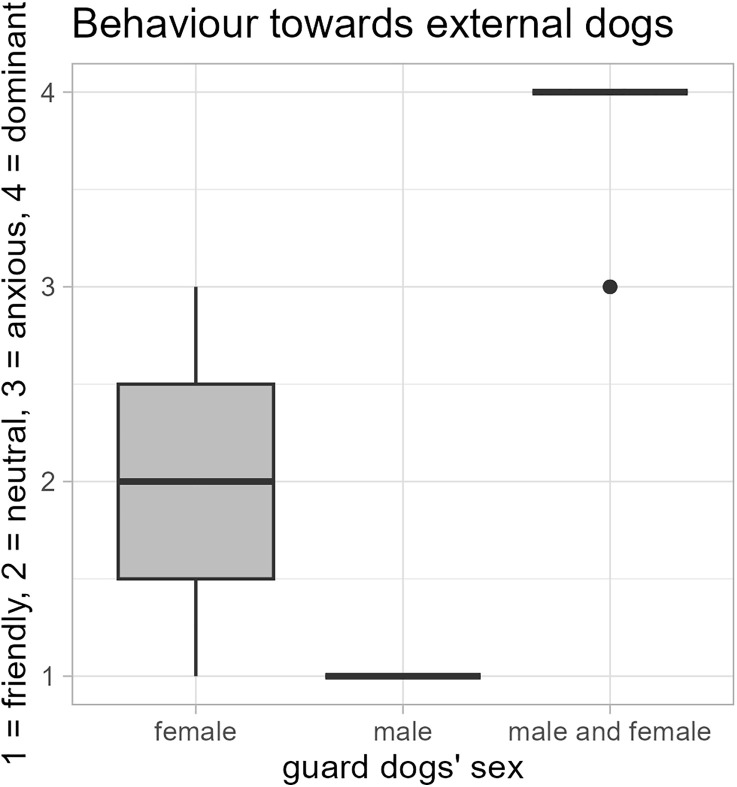
Sex of guard dogs and their behaviour towards external dogs. The experts reported that groups of exclusively male guard dogs (N = 2) were friendly towards external dogs, groups of exclusively female dogs (N = 3) were mostly neutral towards external dogs, and mixed sex groups with male and female guard dogs (N = 20) were dominant towards external dogs (GLM: *N* = 25, *z* = 2.65, *p* = 0.008).

The sex of the guard dog groups affected their behaviour towards other dogs (GLM: *N* = 25, *z* = 2.65, *p* = 0.008). While only female guard dog groups (N = 3) were friendly-attentive, only male groups (N = 2) were friendly and anxious, mixed sex groups (N = 20) were dominant-aggressive towards other dogs (Fig 8).

None of the other factors was reported to have an effect on the guard dogs’ behaviour at the farms (N = 25). These factors encompassed which observer reported the behaviour, the location the guard dogs stayed on farm, the size of the guard dog groups, the number of animals they guarded, the number of groups of animals they guarded, the number of species that lived on the farm, how long the owner kept guard dogs, whether they were bought for wolf protection, the tasks they had, and whether their tasks changed, how the dogs were socialised, the guard dogs age, sex, whether they were intact or neutered or their breed (GLM, *N* = 25, all *p* > 0.05; S3 File).

## Discussion

In direct field observation, the present study found guard dogs preferentially staying within one farm animal body length, rather than in direct body contact or within a distance of one to three body lengths, of goats and horses. This may indicate that guard dogs were well habituated and attached to both species, as reported for dogs guarding free-ranging horses in Iberia [39], and stayed close to them [9,26,29], but were also aware of the danger of potential aggressions by protected species if they were in close, direct body contact [27,30].

The observed guard dogs displayed less anxious and more friendly, neutral and dominant behaviour, which is characteristic for guard dog breeds [26] and needed for the effective protection of the livestock [10,26] and themselves [13,30] if attacked by predators. Guard dogs on German farms were observed to display mostly neutral and friendly behaviour towards their own species, all behaviour categories towards the goats and horses, and mostly dominant behaviour towards external persons, dogs, cats, deer, birds, bikers, and cars, which supports earlier findings that guard dogs were effective and reliable in other countries [9,10,26]. The experts reported that guard dogs displayed the most friendly-attentive behaviour towards their owner and known persons in the presence of the owner, then towards farm animals, and then towards known persons in the absence of the owner and external dogs. Guard dogs were reported to show dominant and aggressive behaviour towards external dogs and unknown persons in the absence of the owner, which is in line with previous behaviour descriptions of guard dogs [26]. Although the expert reports of dogs being dominant and aggressive towards external parties may have been shaped by public expectations [27], they were, nevertheless, supported by the observed dominant behaviour towards external parties in direct field observation. However, guard dogs in the present study displayed dominant and aggressive warning behaviour, but never attacked. They were all well socialised to humans and it was always possible to control them [26]. Therefore, for the studied guard dogs, the public debate of whether it is safe to pass a guarded farm animal group, especially for a child or a domestic dog [27,31], was irrelevant.

The 25 farms included the whole range of guard dog ages and management systems reported in literature [26,28,29,31]. The reported difference between farms concerning the frequency with which guard dogs displayed anxious behaviour may have been a result of the dogs’ management. Guard dogs stayed less in the proximity of one to three farm animal body lengths, and displayed more neutral behaviour, as the size of the guarded animal groups increased, but less neutral behaviour with increasing size of the area. This may simply indicate that they did not attach to individual animals in the larger guarded groups and had to be more watchful when controlling large areas [10,27]. Interestingly, guard dogs preferred to stay within one farm animal body length to the guarded species. However, while they were observed less frequently in the proximity of one to three body lengths, when they were in this proximity, they displayed more dominant behaviour the better they were socialised. Furthermore, older animals exhibited a greater frequency of friendly, anxious, and dominant behaviour than juveniles. Both findings may support the arguments that older and more socialised guard dogs did not attach to specific animals but rather to the farm, and protected their farms and livestock well [26]. This underlines the need to socialise the dogs at least until they are two years old [10,30].

Furthermore, the majority of farms kept mixed sex guard dog groups. However, guard dogs in the two only male and in the three only female groups were reported to show friendly or neutral behaviour in contact with external dogs, while guard dogs in the 20 mixed sex groups were dominant and occasionally anxious. Groups including both intact and neutered animals in mixed sex guard dog groups were observed in this study, but whether the animals were intact or neutered was not reported to make any difference to their work and management [10]. This finding may confirm reports that dogs in single sex groups may search for dogs of the other sex, especially in the mating season [10,26]. Dogs in mixed sex groups may form cohesive and more self-reliant groups [26], as the dogs in mixed sex groups of the present study demonstrated rather dominant behaviour towards external dogs. On a farm, large guard dog groups may develop stable dog packs, and the larger the guard dog groups were, the less the guard dogs searched for direct contact with goats and horses, but rather stayed in the distance of one or one to three body lengths of the guarded animals and displayed more friendly behaviour [27].

In the present study, most farms kept Great Pyreneen guard dogs, while the breeds Kangal, Owtscharkar, and Mastino Españiol, Maremmano Abruzzese, and mixed breeds were less frequent. The reported guard dog breeds differed in the proximity to and behaviour towards livestock, with Great Pyreneen and Kangals staying less often, and Owtscharkar, Owtscharkar-Kangal Mix and Sarplaninac guard dogs more frequently, in a proximity of one to three body lengths of goats and horses, while Great Pyreneen displayed most neutral and anxious behaviour. The breed differences should be treated with caution because of the overrepresentation of Great Pyreneen guard dogs in this study. However, the behaviour and proximity findings may support earlier reports of Kangals, bred for protecting the livestock of whole villages against several large predators independently, being an active, and Great Pyrenees, bred for protecting livestock groups in cooperation with shepherds, a rather adaptive, guard dog breed [26].

Interestingly, other than the sex of the guard dogs, none of the other factors had an effect on the direction of guard dogs’ behaviour at the farms. These factors included the guard dogs’ age, breed, whether they were of intact sex or neutered, the location they stayed on the farm, the size of the guard dog group, the number of animals they guarded, the number of species that lived on the farm, how the dogs were socialised, how long the owner kept guard dogs, the tasks they had, and whether their tasks changed. This finding may support reports of the adaptability of guard dogs to the challenges of any particular farm [9,27,29]. However, further studies should observe individual guard dogs and individual duties in more detail. The experts of the present study, for example, did not report on the socialization of individual guard dogs, which may be crucial for analysing the suitability of individual dog breeds, and the dogs’ age and sex, for particular tasks. Furthermore, future studies should give attention to whether guard dog behaviour might change when the tasks of the dogs’ change. In general, guard dogs preferentially stayed within one farm animal’s body length to the guarded animals. However, we found that they stayed less often in the proximity of one to three guarded animal body lengths and showed less neutral behaviour when the behaviour was recorded with cameras rather than when it was documented by persons in direct field observation. This disparity may have been due to the differences in the presence of observing persons or to the different animal species guarded [26] because goats were observed with camera documentations and horses with personal direct field observations.

Limitations of the study arose from the applied method. Recollection protocols may suffer from recall biases, as they may be hampered by the respondents’ episodic memory [33,40] and their adaptations to public expectations [27,41]. Furthermore, the presence of observers may have changed the observed animals’ behaviour [32], even though we were careful to start the data recording only when the animals ignored the observers. Finally, the recent study conducted field observations only on goats and horses. Even though multiple farm animals were covered by the experts’ reports, future studies may expand also the field observations to further species for a comprehensive insight into guard dog behaviour [9,27].

## Conclusion

Guard dogs were reliable and suitable for duties on German farms [31], especially when well socialised and kept in mixed sex guard dog groups. They adapted to a large variety of tasks at the farms and were observed attaching to goats and horses. They were friendly with the owner of the farm and with known persons, but dominant and watchful against external individuals, such as unknown persons and external dogs. The issue of potential confrontations between a dominant, guarding dog and external persons remains the greatest challenge and requires better communication between guard dog owners and the public. This is currently only undertaken on private initiative, but may need some governmental assistance [12,27].

## Supporting information

S1 Table Field observation data. (XLSX)

S2 Table Protocol data. (XLSX)

S1 File Protocol sheet. An original example of the recollection protocol in German. The translation of the recollection protocol questions in English is given below. The protocol was used to ask experts to report their experiences with guard dogs during their first visits to farms in the preceding year. All original recollection protocols are stored at the server of Nürtingen-Geislingen University and can be viewed on request.
(PDF)

S2 File Inter rater reliability assessment for protocols. Spearman rank correlation analysis for categorical data was used to assess the comparability of multiple recollection protocols for the farms F1-F5. We received 3 protocols for farms F1 – F3, 4 protocols for farm F4 and 2 protocols for farm F5.
(PDF)

S3 File Statistical data. Full data of the statistical analyses of a) the field observation and b) the protocols.
(PDF)

## References

[1] Jain V, Bugnyar T, Cunningham SJ, Gallego-Abenza M, Loretto M-C, Sumasgutner P. The spatial and temporal exploitation of anthropogenic food sources by common ravens (*Corvus corax*) in the Alps. Mov Ecol. 2022;10(1):35. doi: 10.1186/s40462-022-00335-4 36008849 PMC9414151

[2] Spassov N, Acosta-Pankov I. Dispersal history of the golden jackal (*Canis aureus moreoticus Geoffroy*, 1835) in Europe and possible causes of its recent population explosion. Biodivers Data J. 2019;7:e34825. doi: 10.3897/BDJ.7.e34825 31139003 PMC6522460

[3] Kaczensky P, Chapron G, Von Arx M, Huber D, Andrén H, Linnell J. Status, management and distribution of large carnivores-bear, lynx, wolf & wolverine-in Europe. Freiburg. 2013.

[4] Chapron G, Kaczensky P, Linnell JDC, von Arx M, Huber D, Andrén H, et al. Recovery of large carnivores in Europe’s modern human-dominated landscapes. Science. 2014;346(6216):1517–9. doi: 10.1126/science.1257553 25525247

[5] Penteriani V, Huber D, Jerina K, Krofel M, López-Bao JV, Ordiz A, et al. Trans-boundary and trans-regional management of a large carnivore: Managing brown bears across national and regional borders in Europe. Large Carnivore Conservation and Management. Routledge; 2018. pp. 291–313.

[6] Mueller SA, Prost S, Anders O, Breitenmoser-Würsten C, Kleven O, Klinga P, et al. Genome-wide diversity loss in reintroduced Eurasian lynx populations urges immediate conservation management. Biol Conserv. 2022;266:109442. doi: 10.1016/j.biocon.2021.109442

[7] Linnell JDC, Swenson JE, Anderson R. Predators and people: conservation of large carnivores is possible at high human densities if management policy is favourable. Anim Conserv. 2001;4(4):345–9. doi: 10.1017/s1367943001001408

[8] Trouwborst A. Managing the Carnivore Comeback: International and EU Species Protection Law and the Return of Lynx, Wolf and Bear to Western Europe. J Environ Law. 2010;22(3):347–72. doi: 10.1093/jel/eqq013

[9] Allen LR, Stewart-Moore N, Byrne D, Allen BL. Guardian dogs protect sheep by guarding sheep, not by establishing territories and excluding predators. Anim Prod Sci. 2016;57(6):1118–27. doi: 10.1071/an16030

[10] Andelt WF, Hopper SN. Livestock guard dogs reduce predation on domestic sheep in Colorado. J Range Manag. 2000;53(3):259. doi: 10.2307/4003429

[11] Kaczensky P. Large Carnivore – Livestock Conflicts in Europe. NINA Studie. Wildbiologische Gesellschaft München; 1996. pp. 106.

[12] Barmoen M, Bærum KM, Mathiesen KE. Living with wolves: a worldwide systematic review of attitudes. Ambio. 2024;53(10):1414–32. doi: 10.1007/s13280-024-02036-1 38833186 PMC11383909

[13] DBBW. Zusammenstellung der wolfsverursachten Schäden, Präventions- und Ausgleichszahlungen in Deutschland 2023 nach den Angaben der Bundesländer. Berlin: DBBW; 2024.

[14] Council Directive (EEC). 1992. 92/43 of 21 May 1992 on the conservation of natural habitats and of wild fauna and flora; 1979.

[15] Trouwborst A. Large carnivores and the EU habitat directive – legal obligations to restore and coexist. CPDnews. 2025;30:9–15.

[16] Reinhardt I, Kluth G, Nowak C, Szentiks CA, Krone O, Ansorge H, et al. Military training areas facilitate the recolonization of wolves in Germany. Conserv Lett. 2019;12(3). doi: 10.1111/conl.12635

[17] Figari H, Skogen K. Social representations of the wolf. Acta Sociol. 2011;54(4):317–32. doi: 10.1177/0001699311422090

[18] Chapron G, López-Bao JV. Coexistence with large carnivores informed by community ecology. Trends Ecol Evol. 2016;31(8):578–80. doi: 10.1016/j.tree.2016.06.003 27377602

[19] Slagle KM, Wilson RS, Bruskotter JT, Toman E. The Symbolic wolf: a construal level theory analysis of the perceptions of wolves in the United States. Soc Nat Resour. 2018;32(3):322–37. doi: 10.1080/08941920.2018.1501525

[20] Heywood VH, Watson RT. Global biodiversity assessment. Cambridge: Cambridge University Press; 1995

[21] Smith DW, Bangs EE. Reintroduction of wolves to Yellowstone National Park: History, values, and ecosystem restoration. In: Hayward MW, Somers MJ, editors. Reintroduction of top-order predators. Oxford: Wiley-Blackwell; 2009. pp. 92–125.

[22] Linnell JDC, Cretois B. Research for AGRI Committee – The revival of wolves and other large predators and its impact on farmers and their livelihood in rural regions of Europe. Brussels: European Parliament, Policy Department for Structural and Cohesion Policies; 2018.

[23] BfN. Empfehlungen zum Schutz von Weidetieren und Gehegetieren vor dem Wolf. Konkrete Anforderungen an die empfohlenen Präventionsmaßnahmen. BfN-Skripten 530. 2019. pp 14.

[24] van Bommel L, Johnson CN. Still a good dog! Long-term use and effectiveness of livestock guardian dogs to protect livestock from predators in Australia’s extensive grazing systems. Wildl Res. 2023;51(1). doi: 10.1071/wr23008

[25] Coppinger R, Coppinger L. Interaction between livestock guarding dogs and wolves. In: Carbyn LN, Fritts SH, Seip DR, editors. Wolves in a changing world. Edmonton, Alberta, Canada: Canadian Circumpolar Institute; 1995. pp. 523–6.

[26] Landry JM. The use of guard dogs in the Swiss Alps: A first analysis. KORA Bericht Nr. 2 e. ISSN: 1422-5123. 1999

[27] Adams GJ, Johnson KG. Guard dogs: sleep, work and the behavioral responses to people and other stimuli. Appl Anim Behav Sci. 1995;46:103–15.

[28] Coppinger L. Dog performance report 1991. DogLog. 1992;2(3–4).

[29] Coppinger L. Getting through that juvenile period. DogLog. 1992;2:6–12.

[30] Arnold I. Wissen. Lernen. Anders machen - die Rückkehr der Wölfe als Lernprozess. HJK. 2021;13:317–27.

[31] Coppinger L, Coppinger R. Dogs for herding and guarding livestock. Livestock handling and transport. GB: CABI; 2024. pp. 296–313.

[32] Bateson M, Martin P. Measuring behavior: an introductory guide. Cambridge: Cambridge University Press; 2021.

[33] Bryman A. Integrating quantitative and qualitative research: how is it done? Qual Res. 2006;6(1):97–113. doi: 10.1177/1468794106058877

[34] Wolter R, Stefanski V, Krueger K. Parameters for the analysis of social bonds in horses. Animals (Basel). 2018;8(11):191. doi: 10.3390/ani8110191 30373257 PMC6262610

[35] Scott JP, Fuller JL. Genetics and the social behavior of the dog. Chicago: University of Chicago Press; 1998.

[36] Willis M. Genetic aspects of dog behavior with particular reference to working ability. The Domestic Dogs: Its Evolution, Behavior, and Interaction with People. Cambridge: Cambridge University Press; 1995. pp. 51–64.

[37] Akaike H. Information theory and an extension of the maximum likelihood principle. New York: Springer Science and Business Media; 1998.

[38] Holm SA. simple sequentially rejective multiple test procedure. Scand Stat Theory Appl. 1979;6:65–70. https://www.jstor.org/stable/4615733

[39] Lagos L, Blanco P. Testing the use of dogs to prevent wolf attacks on free-ranging ponies in NW Iberia. CPDNews. 2021:20–7.

[40] Brainerd CJ, Gomes CFA, Nakamura K. Dual recollection in episodic memory. J Exp Psychol Gen. 2015;144(4):816–43. doi: 10.1037/xge0000084 26053931

[41] Aden RC, Han MW, Norander S, Pfahl ME, Pollock TP Jr, Young SL. Re-collection: a proposal for refining the study of collective memory and its places. Commun Theory. 2009;19(3):311–36. doi: 10.1111/j.1468-2885.2009.01345.x

