# Supplementary material for: Guard dog behaviour (Canis lupus familiaris) towards various animal species and humans on farms in Germany
Source: PLoS One. 2025 Nov 25;20(11):e0337432. doi: 10.1371/journal.pone.0337432 (PMC12646397; doi:10.1371/journal.pone.0337432)
Supplement: S1 File — An original example of the recollection protocol in German. The translation of the recollection protocol questions in English is given below. The protocol was used to ask experts to report their experiences with guard dogs during their first visits to farms in the preceding year. All original recollection protocols are stored at the server of Nürtingen-Geislingen University and can be viewed on request. (PDF) [file pone.0337432.s003.pdf]

Supporting Information

Guard dog behaviour towards various animal species, and towards internal and external persons, on farms in Germany

Guard dog behaviour on farms in Germany

Konstanze Krueger[1,2,*], Kimberly Scarlet Camenzind[1], Aida Kumpf[2], Kate Farmer[3], Maren Bernau[1]

[1]Department of Equine Economics, Faculty of Agriculture, Economics and Management, Nuertingen-Geislingen University, Neckarsteige 6-10, 72622 Nürtingen, Germany
[2] Zoology/Evolutionary Biology, University of Regensburg, Universitätsstraße 31, 93053 Regensburg, Germany
[3]Centre for Social Learning & Cognitive Evolution, School of Psychology, University of St Andrews, St Andrews, Scotland KY16 9JPh, UK.
* Corresponding author: Konstanze Krueger
Email: Konstanze.krueger@hfwu.de

S1 file. Protocol sheet. a) An original example of the recollection protocol in German. **b**) The translation of the recollection protocol questions in English is given below. The protocol was used to ask experts to report their experiences with guard dogs during their first visits to farms in the preceding year. All original recollection protocols are stored at the server of Nürtingen-Geislingen University and can be viewed on justified request.

a) Original recollection protocol for first visit on farm

Gedächtnisprotokoll zum Ersten Besuch auf dem Betrieb

1. **Wo liegt der Betrieb?**

- [ ] Baden-Württemberg
- [x] Bayern
- [ ] Berlin
- [ ] Brandenburg
- [ ] Bremen
- [ ] Hamburg
- [ ] Hessen
- [ ] Mecklenburg-Vorpommern
- [ ] Niedersachsen
- [ ] Nordrhein-Westfalen
- [ ] Rheinland-Pfalz
- [ ] Saarland
- [ ] Sachsen
- [ ] Sachsen-Anhalt
- [ ] Schleswig-Holstein
- [ ] Thüringen

2. **Wie viele HSH hat der Betrieb?**

- [ ] 1
- [x] 2
- [ ] 3
- [ ] 4
- [ ] 5
- [ ] 6 - 10
- [ ] mehr als 10

3. **Welche HSH-Rassen findet man auf dem Betrieb?** (Mehrfachantworten möglich)

- [ ] Aidi
- [ ] Akbash
- [ ] Cao da Serra da Estrela
- [ ] Cao de Castro Laboreiro
- [ ] Ciobanesc Romanesc Carpatin
- [ ] Ciobanesc Romanesc Mioritic
- [ ] Do Khyi
- [ ] Hellenikos Pimenikos
- [x] Kangal
- [ ] Karakachan
- [ ] Kaukasischer Owtscharka
- [ ] Kommondor
- [ ] Kraski Ovcar
- [ ] Kuvasz
- [ ] Mastin Español
- [ ] Mastin del Pirineo
- [ ] Maremmano Abruzzese
- [ ] Moskauer Wachhund
- [ ] Polski Owczarek Podhalański
- [ ] Pyrenäenberghund
- [ ] Rafeiro do Alentejo
- [ ] Sarplaninac
- [ ] Slovensky Cuvac
- [ ] Südrussischer Owtscharka
- [ ] Tornjak
- [ ] Zentralasiatischer Owtscharka
- [ ] Andere:

4. **Welches Geschlecht haben die HSH auf dem Betrieb?** (Mehrfachantworten möglich)

- [ ] Hündin, nicht kastriert
- [ ] Hündin, kastriert
- [x] Hündin, ohne Angabe zur Kastration
- [ ] Rüde, nicht kastriert
- [ ] Rüde, kastriert
- [ ] Rüde, ohne Angabe zur Kastration

5. **Wie alt sind die HSH auf dem Betrieb?** (Mehrfachantworten möglich)
   - ☐ unter 6 Monaten
   - ☐ 5 – 10 Jahre
   - ☐ 6 Monate bis 1 Jahr
   - ☐ Älter als 11 Jahre
   - ☐ 1 – 5 Jahre

6. **In welchem Arbeitsbereich werden die HSH eingesetzt?** (Mehrfachantworten möglich)
   - ☒ Herdenschutzhund in der Herde
   - ☐ Haushund
   - ☒ Wach- und Schutzhund auf dem Hof
   - ☐ Andere:

7. **Wechseln die Arbeitsbereiche in denen die HSH eingesetzt werden?**
   - ☐ Ja, regelmäßig
   - ☒ Nein
   - ☐ Ja, unregelmäßig

8. **Wurden die HSH als Wolfsschutz angeschafft?**
   - ☒ Ja
   - ☐ Nein

9. **Wie lange besitzt der Hundehalter schon HSH?**
   - ☐ weniger als 1 Jahr
   - ☐ 6 – 10 Jahre
   - ☐ 1 – 5 Jahre
   - ☐ mehr als 10 Jahre

10. **Welche Tierarten gibt es auf dem Hof und in welcher Anzahl?** (Mehrfachantworten möglich)
    - ☒ Schafe, Anzahl:
    - ☐ Hühner, Anzahl:
    - ☐ Ziegen, Anzahl:
    - ☐ Enten, Anzahl:
    - ☒ Pferde, Anzahl:
    - ☐ Kaninchen, Anzahl:
    - ☐ Esel, Anzahl:
    - ☐ Katzen, Anzahl:
    - ☐ Kühe, Anzahl:
    - ☒ Andere Hunde, Anzahl: 1
    - ☐ Schweine, Anzahl:
    - ☐ Andere: Anzahl:

11. **Wie viele Tiergruppen wurden von den HSH bewacht?**
    - ☐ 1 Gruppe, Welche Tierart:
    - ☒ 2 Gruppen, Welche Tierarten: Schafe und Pferde
    - ☐ 3 Gruppen, Welche Tierarten:
    - ☐ 4 Gruppen, Welche Tierarten:
    - ☐ 5 Gruppen, Welche Tierarten:
    - ☐ 5 – 10 Gruppen, Welche Tierarten:
    - ☐ Mehr als 10 Gruppen, Anzahl: Welche Tierarten:

12. **Wie reagieren die HSH auf neue/fremde Hunde?** (Mehrfachantworten möglich)

- [ ] keine Reaktion
- [ ] wedeln
- [x] beschnüffeln
- [ ] hält Abstand
- [ ] zieht Kopf und Schwanz ein
- [ ] weicht aus
- [ ] macht sich groß
- [x] sucht Kontakt
- [ ] Bellen
- [ ] Knurren
- [ ] läuft hin und her

13. **Wie reagieren die HSH auf neue/fremde Menschen ohne den Besitzer?** (Mehrfachantworten möglich)

- [ ] keine Reaktion
- [ ] wedeln
- [x] beschnüffeln
- [ ] hält Abstand
- [ ] zieht Kopf und Schwanz ein
- [ ] weicht aus
- [ ] macht sich groß
- [x] sucht Kontakt
- [ ] Bellen
- [ ] Knurren
- [ ] läuft hin und her

14. **Wie reagieren die HSH auf neue/ fremde Menschen in Anwesenheit des Besitzers?** (Mehrfachantworten möglich)

- [ ] keine Reaktion
- [ ] wedeln
- [x] beschnüffeln
- [ ] hält Abstand
- [ ] zieht Kopf und Schwanz ein
- [ ] weicht aus
- [ ] macht sich groß
- [x] sucht Kontakt
- [ ] Bellen
- [ ] Knurren
- [ ] läuft hin und her

15. **Wie reagieren die HSH auf andere (am Hof lebende) Tiere?** (Mehrfachantworten möglich)

- [ ] keine Reaktion
- [ ] wedeln
- [ ] beschnüffeln
- [ ] hält Abstand
- [ ] zieht Kopf und Schwanz ein
- [ ] weicht aus
- [ ] macht sich groß
- [ ] sucht Kontakt
- [ ] Bellen
- [ ] Knurren
- [ ] läuft hin und her

16. **Wie reagieren die HSH auf den Besitzer?** (Mehrfachantworten möglich)

- [ ] keine Reaktion
- [x] wedeln
- [ ] beschnüffeln
- [ ] hält Abstand
- [ ] zieht Kopf und Schwanz ein
- [ ] weicht aus
- [ ] macht sich groß
- [x] sucht Kontakt
- [ ] Bellen
- [ ] Knurren
- [ ] läuft hin und her

17. **Wie reagieren die HSH auf bekannte Menschen ohne den Besitzer?** (Mehrfachantworten möglich)

- ☐ keine Reaktion
- ☐ wedeln
- ☐ beschnüffeln
- ☐ hält Abstand
- ☐ zieht Kopf und Schwanz ein
- ☐ weicht aus
- ☐ macht sich groß
- ☐ sucht Kontakt
- ☐ Bellen
- ☐ Knurren
- ☐ läuft hin und her

18. **Wie reagieren die HSH auf bekannte Menschen in Anwesenheit des Besitzers?** (Mehrfachantworten möglich)

- ☐ keine Reaktion
- ☐ wedeln
- ☐ beschnüffeln
- ☐ hält Abstand
- ☐ zieht Kopf und Schwanz ein
- ☐ weicht aus
- ☐ macht sich groß
- ☐ sucht Kontakt
- ☐ Bellen
- ☐ Knurren
- ☐ läuft hin und her

19. **Wo halten sich die HSH in der Regel auf?**

- ☐ In der Umzäunung, in der Herde
- ☐ In der Umzäunung abseits der Herde
- ☐ In der Nähe der Herde in einem begrenzten Bereich
- ☒ Auf dem Hof
- ☐ Auf dem Hof in einem begrenzten Bereich
- ☒ Im Haus
- ☐ Beim Besitzer
- ☐ Anderer Ort:

20. **Hauen die HSH ab, wenn sie die Möglichkeit dazu haben?**

- ☐ Ja, regelmäßig (Wöchentlich)
- ☐ Ja, ab und zu (Monatlich)
- ☐ Ja, selten (Jährlich)
- ☒ Nein Vor Erziehung häufiger

21. **Sind die HSH bei Ankunft der Studenten da, wenn nicht, wann kommen sie dazu?**

- ☐ Ja
- ☐ Nein, aber innerhalb von 1min
- ☐ Nein, aber innerhalb von 5min
- ☐ Nein, aber innerhalb von 10min
- ☒ Nein, aber sobald der Besitzer den Besuch begrüßt Waren vorher im Haus
- ☐ Nein, gar nicht

22. Bisheriges Handling/Sozialisierung der HSH? (Mehrfachantworten möglich)

- ☐ Schon als Welpen bekommen
- ☐ Erst als erwachsenes Tier angeschafft
- ☐ Welpen wurden getrennt von Hofgeschehen aufgezogen
- ☐ Welpen wurden ohne Einfluss des Menschen aufgezogen
- ☐ Welpen hatten nur Kontakt mit Besitzer
- ☐ Welpen wurde mit Tieren bekannt gemacht, Welche Tiere:
- ☐ Welpen wurden mit Kindern bekannt gemacht
- ☐ Welpen wurden mit fremden Menschen bekannt gemacht
- ☒ HSH lernen regelmäßig neue Menschen kennen Besucher
- ☐ HSH haben regelmäßig Kontakt mit Kindern
- ☒ HSH haben regelmäßig Kontakt mit bekannten Menschen, die nicht Besitzer sind Einsteller
- ☐ HSH haben nur Kontakt mit Besitzer

23. Weitere Ergänzungen (z.b. Erzählungen der Besitzer):

b) Translation of the protocol questions

1. In which area is the farm located?
Baden-Württemberg
Bavaria
Berlin
Brandenburg
Bremen
Hamburg
Hesse
Mecklenburg-Western Pomerania
Lower Saxony
North Rhine-Westphalia
Rhineland-Palatinate
Saarland
Saxony
Saxony-Anhalt
Schleswig-Holstein
Thuringia

2. How many guard dogs are on farm?
1, 2, 3, 4, 5, 6 – 10, more than 10

3. Which guard dog breeds were found on farm? (multiple answers possible)
Aidi Akbash
Cao da Serra da Estrela
Cao de Castro Laboreiro
Ciobanesc Romanesc
Carpatin Ciobanesc
Romanesc Mioritic
Do Khyi
Hellenikos Pimenikos
Kangal
Karakachan
Caucasian Ovcharka
Commander
Kraski Ovcar
Kuvasz
Mastin Español
Mastin del Pirineo
Maremmano Abruzzese
Moscow watchdog
Polski Owczarek Podhalański
Great Pyrenees Mountain Dog
Rafeiro do Alentejo
Sarplaninac
Slovensky Cuvac
South Russian Ovcharka
Tornjak
Central Asian Ovcharka
Other:

4. Which sex did the guard dogs have? (multiple answers possible)
Female dog, intact
Female dog, not intact

Female dog, no information about spaying
Male dog, intact
Male dog, not intact
Male dog, no information about spaying

5. How old were the guard dogs? (multiple answers possible)
below 6 months, 6 months up to 1 year, 1 – 5 years, 5 – 10 years, older than 11 years

6. to which task were the guard dogs assigned? (multiple answers possible)
Livestock guardian dog in the herd
Guard and protection dog on the farm
House dog
Other:

7. Did the task of the guard dogs change?
yes, regularly, unregularly, no

8. Were guard dogs bought for protection from wolves?
yes, no

9. Wo long does the owner keep guard dogs?
less than 1 year, 1 – 5 years, 6 – 10 years, more than 10 years

10. Which animal species did you find at the farm in which numbers? (multiple answers possible)
Sheep, Number: ,Goats, Number: ,Horses, Number: ,Donkeys, Number: ,Cows, Number: ,Pigs, Number: ,Chickens, Number: ,Ducks, Number: ,Rabbits, Number: ,Cats, Number: ,Other Dogs, Number: ,Other: Number:

11. How many animal groups were guarded by the dogs?
1 group, which animal species:
2 groups, which animal species:
3 groups, which animal species:
4 groups, which animal species:
5 groups, which animal species:
5 – 10 groups, which animal species:
More than 10 groups, number: which animal species:

12. How did guard dogs respond to new / unknown dogs? (multiple answers possible)
no reaction, wagging, sniffing, keeping distance, pulling head and tail in, avoiding, making himself big, looking for contact, barking, growling, running back and forth

13. How did guard dogs respond to new / unknown persons in the absence of the owner? (multiple answers possible)
no reaction, wagging, sniffing, keeping distance, pulling head and tail in, avoiding, making himself big, looking for contact, barking, growling, running back and forth

14. How did guard dogs respond to new / unknown persons in the presence of the owner? (multiple answers possible)
no reaction, wagging, sniffing, keeping distance, pulling head and tail in, avoiding, making himself big, looking for contact, barking, growling, running back and forth

15. How did guard dogs respond to other animals on the farm? (multiple answers possible)
no reaction, wagging, sniffing, keeping distance, pulling head and tail in, avoiding, making himself big, looking for contact, barking, growling, running back and forth

16. How did guard dogs respond to the owner? (multiple answers possible)
no reaction, wagging, sniffing, keeping distance, pulling head and tail in, avoiding, making himself big, looking for contact, barking, growling, running back and forth

17. How did guard dogs respond to known persons in the absence of the owner?
(multiple answers possible)
no reaction, wagging, sniffing, keeping distance, pulling head and tail in, avoiding, making himself big, looking for contact, barking, growling, running back and forth

18. How did guard dogs respond to known persons in the presence of the owner?
(multiple answers possible)
no reaction, wagging, sniffing, keeping distance, pulling head and tail in, avoiding, making himself big, looking for contact, barking, growling, running back and forth

19. Where do guard dogs stay at the farms?
In the enclosure, in the herd,
In the enclosure away from the herd,
Near the herd in a confined area,
In the yard,
In the yard in a confined area,
In the house,
At the owner's home,
Other location:

20. Do guard dogs run away?
Yes, often (every week), yes occasionally (once a month), yes rarely (once a year), no

21. Were guard dogs present when you arrived? If not, did they join you later?
Yes
No, but within 1 minute
No, but within 5 minutes
No, but within 10 minutes
No, but as soon as the owner greets the visitor
No, not at all

22. Did the guard dogs receive handling / socialisation? (multiple answers possible)
Acquired as puppies
Purchased only as adults
Puppies were raised separately from farm activities
Puppies were raised without human influence
Puppies only had contact with their owners
Puppies were introduced to animals
Which animals: Puppies were introduced to children
Puppies were introduced to strangers.
Guard dogs regularly meet new people
Guard dogs have regular contact with children
Guard dogs have regular contact with familiar people who are not their owners
Guard dogs only have contact with their owners

23. Further additions (e.g. stories from the owners):
