# Supplementary material for: Guard dog behaviour (Canis lupus familiaris) towards various animal species and humans on farms in Germany
Source: PLoS One. 2025 Nov 25;20(11):e0337432. doi: 10.1371/journal.pone.0337432 (PMC12646397; doi:10.1371/journal.pone.0337432)
Supplement: S2 File — Spearman rank correlation analysis for categorical data was used to assess the comparability of multiple recollection protocols for the farms F1-F5. We received 3 protocols for farms F1 – F3, 4 protocols for farm F4 and 2 protocols for farm F5. (PDF) [file pone.0337432.s004.pdf]

Supporting Information

Guard dog behaviour towards various animal species, and towards internal and external persons, on farms in Germany

Guard dog behaviour on farms in Germany

Konstanze Krueger[1,2,*], Kimberly Scarlet Camenzind[1], Aida Kumpf[2], Kate Farmer[3], Maren Bernau[1]

[1]Department of Equine Economics, Faculty of Agriculture, Economics and Management, Nuertingen-Geislingen University, Neckarsteige 6-10, 72622 Nürtingen, Germany
[2] Zoology/Evolutionary Biology, University of Regensburg, Universitätsstraße 31, 93053 Regensburg, Germany
[3]Centre for Social Learning & Cognitive Evolution, School of Psychology, University of St Andrews, St Andrews, Scotland KY16 9JPh, UK.
* Corresponding author: Konstanze Krueger
Email: Konstanze.krueger@hfwu.de

S2 File. Inter rater reliability assessment for protocols. Spearman rank correlation analysis for categorical data was used to assess the comparability of multiple recollection protocols for the farms F1-F5. We received 3 protocols for farms F1 – F3, 4 protocols for farm F4 and 2 protocols for farm F5.

Spearman correlations farm F1:

	P1	P2	P3
P1	1.0000	0.5905	0.6513
P2	0.5905	1.0000	0.8868
P3	0.6513	0.	

Pairwise two-sided p-values:

	P1	P2	P3
P1		0.0048	0.0014
P2	0.0048		<.0001
P3	0.0014	<.0001	

Spearman correlations farm F2:

	P4	P5	P6
P4	1.0000	0.9453	0.8217
P5	0.9453	1.0000	0.8963
P6	0.8217	0.8963	1.0000

Pairwise two-sided p-values:

	P4	P5	P6
P4		<.0001	<.0001
P5	<.0001		<.0001
P6	<.0001	<.0001	

Spearman correlations farm F3:

	P7	P8	P9
P7	1.0000	0.8954	0.9035
P8	0.8954	1.0000	0.9526
P9	0.9035	0.9526	1.0000

Pairwise two-sided p-values:

	P7	P8	P9
P7		<.0001	<.0001
P8	<.0001		<.0001
P9	<.0001	<.0001	

Spearman correlations farm F4:

	P10	P11	P12	P13
P10	1.0000	0.7357	0.9268	0.9565
P11	0.7357	1.0000	0.6895	0.7638
P12	0.9268	0.6895	1.0000	0.9404
P13	0.9565	0.7638	0.9404	1.0000

Pairwise two-sided p-values:

	P10	P11	P12	P13
P10		0.0001	<.0001	<.0001
P11	0.0001		0.0005	<.0001
P12	<.0001	0.0005		<.0001
P13	<.0001	<.0001	<.0001	

Spearman correlations farm F5:

	P14	P15
P14	1.0000	0.9637
P15	0.9637	1.0000

Pairwise two-sided p-values:

	P14	P15
P14		<.0001
P15	<.0001	
