# Supplementary material for: Guard dog behaviour (Canis lupus familiaris) towards various animal species and humans on farms in Germany
Source: PLoS One. 2025 Nov 25;20(11):e0337432. doi: 10.1371/journal.pone.0337432 (PMC12646397; doi:10.1371/journal.pone.0337432)
Supplement: S3 File — Full data of the statistical analyses of a) the field observation and b) the protocols. (PDF) [file pone.0337432.s005.pdf]

Supporting Information

Guard dog behaviour towards various animal species, and towards internal and external persons, on farms in Germany

Guard dog behaviour on farms in Germany

Konstanze Krueger[1,2,*], Kimberly Scarlet Camenzind[1], Aida Kumpf[2], Kate Farmer[3], Maren Bernau[1]

[1]Department of Equine Economics, Faculty of Agriculture, Economics and Management, Nuertingen-Geislingen University, Neckarsteige 6-10, 72622 Nürtingen, Germany
[2] Zoology/Evolutionary Biology, University of Regensburg, Universitätsstraße 31, 93053 Regensburg, Germany
[3]Centre for Social Learning & Cognitive Evolution, School of Psychology, University of St Andrews, St Andrews, Scotland KY16 9JPh, UK.
* Corresponding author: Konstanze Krueger
Email: Konstanze.krueger@hfwu.de

S3 File. Statistical data. Full data of the statistical analyses of **a**) the field observation and **b**) the protocols.

a) statistical analyses of the field observation

Principal Component Analysis proximity (NNA) of guard dogs with guarded animals

```
Rcmdr>  local({
Rcmdr+    .PC <- princomp(~NNA..frequency.in.1.3.body.length.h+NNA.frequency.contact.h+NNA.frequency.in.body.length.h,
cor=TRUE, data=Dataset)
Rcmdr+    cat("\nComponent loadings:\n")
Rcmdr+    print(unclass(loadings(.PC)))
Rcmdr+    cat("\nComponent variances:\n")
Rcmdr+    print(.PC$sd^2)
Rcmdr+    cat("\n")
Rcmdr+    print(summary(.PC))
Rcmdr+  })

Component loadings:
                                        Comp.1     Comp.2     Comp.3
NNA..frequency.in.1.3.body.length.h  0.6358029  0.1947873  0.7468685
NNA.frequency.contact.h              0.4900215 -0.8494815 -0.1956019
NNA.frequency.in.body.length.h      -0.5963503 -0.4903459  0.6355527

Component variances:
   Comp.1    Comp.2    Comp.3 
1.4142627 0.8776288 0.7081086 

Importance of components:
                          Comp.1    Comp.2    Comp.3
Standard deviation     1.1892278 0.9368184 0.8414919
Proportion of Variance 0.4714209 0.2925429 0.2360362
Cumulative Proportion  0.4714209 0.7639638 1.0000000
```

Median comparisons proximity (NNA) of guard dogs with guarded animals

```
Medians:
NNA..frequency.in.1.3.body.length.h             NNA.frequency.contact.h 
                          0.0000000                           0.0000000 
     NNA.frequency.in.body.length.h 
                          0.1904762 

        Friedman rank sum test

data:  .Responses
Friedman chi-squared = 9.8919, df = 2, p-value = 0.007112


        Wilcoxon signed rank test with continuity correction

data:  NNA..frequency.in.1.3.body.length.h and NNA.frequency.contact.h
V = 20, p-value = 0.05778
alternative hypothesis: true location shift is not equal to 0


        Wilcoxon signed rank test with continuity correction

data:  NNA..frequency.in.1.3.body.length.h and NNA.frequency.in.body.length.h
V = 17, p-value = 0.1673
alternative hypothesis: true location shift is not equal to 0


        Wilcoxon signed rank test with continuity correction

data:  NNA.frequency.contact.h and NNA.frequency.in.body.length.h
V = 0, p-value = 0.008969
alternative hypothesis: true location shift is not equal to 0
```

Principal Component Analysis behaviour classes guard dogs undirected

```
Rcmdr>  local({
Rcmdr+    .PC <- princomp(~behaviour..guard.dog...anxious...h+behaviour..guard.dog...dominant.agressive...h+behaviour.
.guard.dog...friendly...attentive...h+behaviour..guard.dog...neutral.relaxed...h, cor=TRUE, data=Dataset)
Rcmdr+    cat("\nComponent loadings:\n")
Rcmdr+    print(unclass(loadings(.PC)))
Rcmdr+    cat("\nComponent variances:\n")
Rcmdr+    print(.PC$sd^2)
Rcmdr+    cat("\n")
Rcmdr+    print(summary(.PC))
Rcmdr+  })

Component loadings:
                                            Comp.1     Comp.2      Comp.3     Comp.4
behaviour..guard.dog...anxious...h       0.4866466  0.4539583  0.73749873  0.1148588
behaviour..guard.dog...dominant.agressive...h   0.4792282 -0.7021831  0.03415993  0.5254640
behaviour..guard.dog...friendly...attentive...h 0.5443813 -0.2322140 -0.09155607 -0.8008390
behaviour..guard.dog...neutral.relaxed...h      0.4869952  0.4969280 -0.66824113  0.2633476

Component variances:
    Comp.1     Comp.2     Comp.3     Comp.4 
3.11000023 0.53785430 0.27884946 0.07329601 

Importance of components:
                          Comp.1    Comp.2     Comp.3    Comp.4
Standard deviation     1.7635193 0.7333855 0.52806198 0.2707324
Proportion of Variance 0.7775001 0.1344636 0.06971236 0.0183240
Cumulative Proportion  0.7775001 0.9119636 0.98167600 1.0000000
```

Median comparisons behaviour classes guard dogs undirected

```
Medians:
             anxious.h   dominant.agressive.h friendly...attentive.h
             0.0952381              0.7619048              1.1000000
     neutral.relaxed.h
             1.0000000


        Friedman rank sum test

data:  .Responses
Friedman chi-squared = 22.429, df = 3, p-value = 0.00005312


        Wilcoxon signed rank test with continuity correction

data:  anxious.h and dominant.agressive.h
V = 3, p-value = 0.003324
alternative hypothesis: true location shift is not equal to 0


        Wilcoxon signed rank exact test

data:  anxious.h and friendly...attentive.h
V = 0, p-value = 0.00006104
alternative hypothesis: true location shift is not equal to 0



        Wilcoxon signed rank test with continuity correction

data:  anxious.h and neutral.relaxed.h
V = 0, p-value = 0.001097
alternative hypothesis: true location shift is not equal to 0


        Wilcoxon signed rank test with continuity correction

data:  dominant.agressive.h and friendly...attentive.h
V = 39.5, p-value = 0.2555
alternative hypothesis: true location shift is not equal to 0
```

```
        Wilcoxon signed rank test with continuity correction

data:  dominant.agressive.h and neutral.relaxed.h
V = 40, p-value = 0.268
alternative hypothesis: true location shift is not equal to 0

        Wilcoxon signed rank exact test

data:  friendly...attentive.h and neutral.relaxed.h
V = 44, p-value = 0.3894
alternative hypothesis: true location shift is not equal to 0
```

Generalized Linear Models (GLMs) proximity (NNA)

```
Call:
glm(formula = NNA.frequency.contact.h ~ age_status_num + breed_num + 
    farm + intact_num + nr_animals.guarded + observation.direct_camera_num + 
    sex_num + size_group + size_guarded_area + socialisation_num, 
    family = gaussian(identity), data = Dataset)

Deviance Residuals: 
      Min         1Q     Median         3Q        Max  
-0.023210  -0.003443   0.000000   0.003443   0.022244  

Coefficients: (1 not defined because of singularities)
                               Estimate Std. Error t value Pr(>|t|)
(Intercept)                   -9.543793   5.580480  -1.710    0.121
age_status_num                -0.013498   0.011061  -1.220    0.253
breed_num                     26.676740  15.509905   1.720    0.120
farm                         -33.485720  19.468856  -1.720    0.120
intact_num                     0.002029   0.009912   0.205    0.842
nr_animals.guarded             0.437542   0.254738   1.718    0.120
observation.direct_camera_num  15.302187   8.922529   1.715    0.120
sex_num                        0.005783   0.008093   0.715    0.493
size_group                     6.464923   3.759224   1.720    0.120
size_guarded_area             -0.007248   0.004215  -1.720    0.120
socialisation_num                    NA         NA      NA       NA

(Dispersion parameter for gaussian family taken to be 0.0002017481)

    Null deviance: 0.0057115  on 18  degrees of freedom
Residual deviance: 0.0018157  on  9  degrees of freedom
AIC: -99.939

Number of Fisher Scoring iterations: 2
```

```
Call:
glm(formula = NNA.frequency.contact.h ~ farm + size_group, family = gaussian(identity),
    data = Dataset)

Deviance Residuals:
      Min         1Q     Median         3Q        Max
-0.022202  -0.007775   0.001123   0.001123   0.030372

Coefficients:
              Estimate Std. Error t value Pr(>|t|)
(Intercept)  0.0017167  0.0107291   0.160   0.8749
farm         0.0065006  0.0032762   1.984   0.0647 .
size_group  -0.0024035  0.0009925  -2.422   0.0277 *
---
Signif. codes:  0 '***' 0.001 '**' 0.01 '*' 0.05 '.' 0.1 ' ' 1

(Dispersion parameter for gaussian family taken to be 0.000251476)

    Null deviance: 0.0057115  on 18  degrees of freedom
Residual deviance: 0.0040236  on 16  degrees of freedom
AIC: -98.821

Number of Fisher Scoring iterations: 2
```

```
Call:
glm(formula = NNA.frequency.in.body.length.h ~ age_status_num +
    breed_num + farm + intact_num + nr_animals.guarded + observation.direct_camera_num +
    sex_num + size_group + size_guarded_area + socialisation_num,
    family = gaussian(identity), data = Dataset)

Deviance Residuals:
     Min        1Q    Median        3Q       Max
-0.37520  -0.02507   0.00591   0.06295   0.19623

Coefficients: (1 not defined because of singularities)
                               Estimate Std. Error t value Pr(>|t|)
(Intercept)                   -78.20734   68.96791  -1.134    0.286
age_status_num                 -0.07000    0.13670  -0.512    0.621
breed_num                     217.46827  191.68347   1.135    0.286
farm                         -272.95076  240.61127  -1.134    0.286
intact_num                     -0.05976    0.12250  -0.488    0.637
nr_animals.guarded              3.57093    3.14825   1.134    0.286
observation.direct_camera_num 125.01788  110.27155   1.134    0.286
sex_num                         0.02869    0.10002   0.287    0.781
size_group                     52.74149   46.45942   1.135    0.286
size_guarded_area              -0.05910    0.05209  -1.135    0.286
socialisation_num                    NA         NA      NA       NA

(Dispersion parameter for gaussian family taken to be 0.03081488)

    Null deviance: 0.72464  on 18  degrees of freedom
Residual deviance: 0.27733  on  9  degrees of freedom
AIC: -4.3928

Number of Fisher Scoring iterations: 2
```

```
Call:
glm(formula = NNA.frequency.in.body.length.h ~ size_group, family = gaussian(identity),
    data = Dataset)

Deviance Residuals:
     Min        1Q    Median        3Q       Max
-0.38509  -0.04720   0.01106   0.08530   0.18634

Coefficients:
             Estimate Std. Error t value Pr(>|t|)
(Intercept) -0.020380   0.057108  -0.357 0.725583
size_group   0.033789   0.007753   4.358 0.000428 ***
---
Signif. codes:  0 '***' 0.001 '**' 0.01 '*' 0.05 '.' 0.1 ' ' 1

(Dispersion parameter for gaussian family taken to be 0.02013126)

    Null deviance: 0.72464  on 18  degrees of freedom
Residual deviance: 0.34223  on 17  degrees of freedom
AIC: -16.398

Number of Fisher Scoring iterations: 2
```

```
Call:
glm(formula = farm ~ age_status_num + breed_num + intact_num + 
    nr_animals.guarded + observation.direct_camera_num + sex_num + 
    size_group + size_guarded_area + socialisation_num, family = gaussian(identity), 
    data = Dataset)

Deviance Residuals: 
       Min          1Q      Median          3Q         Max  
-8.882e-16   0.000e+00   4.441e-16   1.554e-15   3.997e-15  

Coefficients:
                                Estimate Std. Error    t value Pr(>|t|)    
(Intercept)                    2.243e+00  7.830e-12  2.864e+11   <2e-16 ***
age_status_num                -3.611e-15  1.759e-15 -2.053e+00   0.0702 .  
breed_num                      3.469e-01  1.392e-12  2.492e+11   <2e-16 ***
intact_num                    -1.815e-15  1.576e-15 -1.151e+00   0.2792    
nr_animals.guarded            -3.617e-03  5.170e-14 -6.996e+10   <2e-16 ***
observation.direct_camera_num -9.917e-01  4.489e-12 -2.209e+11   <2e-16 ***
sex_num                        2.304e-16  1.287e-15  1.790e-01   0.8618    
size_group                     1.989e-01  1.804e-14  1.103e+13   <2e-16 ***
size_guarded_area             -2.755e-16  6.701e-16 -4.110e-01   0.6907    
socialisation_num             -3.247e-01  1.005e-12 -3.231e+11   <2e-16 ***
---
Signif. codes:  0 '***' 0.001 '**' 0.01 '*' 0.05 '.' 0.1 ' ' 1

(Dispersion parameter for gaussian family taken to be 5.100205e-30)

    Null deviance: 3.0737e+01  on 18  degrees of freedom
Residual deviance: 4.5902e-29  on  9  degrees of freedom
AIC: -1219.8

Number of Fisher Scoring iterations: 1
```

```
Call:
glm(formula = farm ~ breed_num + nr_animals.guarded + observation.direct_camera_num +
    size_group + socialisation_num, family = gaussian(identity),
    data = Dataset)

Deviance Residuals:
      Min         1Q     Median         3Q        Max
0.000e+00  5.551e-16  1.332e-15  1.776e-15  1.776e-15

Coefficients:
                                Estimate Std. Error    t value Pr(>|t|)
(Intercept)                    2.243e+00  3.437e-15  6.526e+14   <2e-16 ***
breed_num                      3.469e-01  1.635e-16  2.122e+15   <2e-16 ***
nr_animals.guarded            -3.617e-03  2.969e-17 -1.218e+14   <2e-16 ***
observation.direct_camera_num -9.917e-01  1.788e-15 -5.547e+14   <2e-16 ***
size_group                     1.989e-01  1.332e-16  1.494e+15   <2e-16 ***
socialisation_num             -3.247e-01  8.639e-16 -3.759e+14   <2e-16 ***
---
Signif. codes:  0 '***' 0.001 '**' 0.01 '*' 0.05 '.' 0.1 ' ' 1

(Dispersion parameter for gaussian family taken to be 2.677576e-30)

    Null deviance: 3.0737e+01  on 18  degrees of freedom
Residual deviance: 3.4808e-29  on 13  degrees of freedom
AIC: -1233.1

Number of Fisher Scoring iterations: 1
```

Generalized Linear Models behaviour undirected

```
Call:
glm(formula = friendly...attentive.h ~ age_status_num + breed_num +
    farm + intact_num + nr_animals.guarded + observation.direct_camera_num +
    sex_num + size_group + size_guarded_area + socialisation_num,
    family = gaussian(identity), data = Dataset)

Deviance Residuals:
    Min       1Q   Median       3Q      Max
-2.8013  -0.3704   0.0000   0.4105   3.1986

Coefficients: (1 not defined because of singularities)
                                Estimate Std. Error t value Pr(>|t|)
(Intercept)                    -28.49592  693.78922  -0.041    0.968
age_status_num                   2.36326    1.37516   1.719    0.120
breed_num                       68.50379 1928.25798   0.036    0.972
farm                           -85.84556 2420.45180  -0.035    0.972
intact_num                       0.49928    1.23230   0.405    0.695
nr_animals.guarded               1.11776   31.67013   0.035    0.973
observation.direct_camera_num   40.36171 1109.28710   0.036    0.972
sex_num                          0.28505    1.00617   0.283    0.783
size_group                      16.99155  467.36290   0.036    0.972
size_guarded_area               -0.01861    0.52401  -0.036    0.972
socialisation_num                     NA         NA      NA       NA

(Dispersion parameter for gaussian family taken to be 3.118325)

    Null deviance: 82.380  on 18  degrees of freedom
Residual deviance: 28.065  on  9  degrees of freedom
AIC: 83.331

Number of Fisher Scoring iterations: 2
```

```
Call:
glm(formula = friendly...attentive.h ~ age_status_num + size_group,
    family = gaussian(identity), data = Dataset)

Deviance Residuals:
    Min       1Q   Median       3Q      Max
-2.8509  -0.6604   0.0666   0.4757   3.7503

Coefficients:
               Estimate Std. Error t value Pr(>|t|)
(Intercept)    -2.06202    0.92336  -2.233 0.040171 *
age_status_num  1.68455    0.77082   2.185 0.044076 *
size_group      0.44115    0.09064   4.867 0.000171 ***
---
Signif. codes:  0 '***' 0.001 '**' 0.01 '*' 0.05 '.' 0.1 ' ' 1

(Dispersion parameter for gaussian family taken to be 2.071938)

    Null deviance: 82.380  on 18  degrees of freedom
Residual deviance: 33.151  on 16  degrees of freedom
AIC: 72.496

Number of Fisher Scoring iterations: 2
```

```
Call:
glm(formula = neutral.relaxed.h ~ age_status_num + breed_num +
    farm + intact_num + nr_animals.guarded + observation.direct_camera_num +
    sex_num + size_group + size_guarded_area + socialisation_num,
    family = gaussian(identity), data = Dataset)

Deviance Residuals:
    Min       1Q   Median       3Q      Max
-3.4119  -0.2774   0.3119   0.4689   1.2548

Coefficients: (1 not defined because of singularities)
                                Estimate  Std. Error t value Pr(>|t|)
(Intercept)                   -68.759583  533.071821  -0.129    0.900
age_status_num                 -0.001828    1.056598  -0.002    0.999
breed_num                     180.079016 1481.573900   0.122    0.906
farm                         -224.390484 1859.750224  -0.121    0.907
intact_num                     -0.111333    0.946838  -0.118    0.909
nr_animals.guarded              2.993334   24.333692   0.123    0.905
observation.direct_camera_num  107.190329  852.318945   0.126    0.903
sex_num                         0.712372    0.773090   0.921    0.381
size_group                     43.457457  359.097528   0.121    0.906
size_guarded_area              -0.049388    0.402621  -0.123    0.905
socialisation_num                     NA          NA      NA       NA

(Dispersion parameter for gaussian family taken to be 1.840932)

    Null deviance: 72.402  on 18  degrees of freedom
Residual deviance: 16.568  on  9  degrees of freedom
AIC: 73.318

Number of Fisher Scoring iterations: 2
```

```
Call:
glm(formula = neutral.relaxed.h ~ breed_num + nr_animals.guarded +
    observation.direct_camera_num + size_guarded_area, family = gaussian(identity),
    data = Dataset)

Deviance Residuals:
    Min       1Q   Median       3Q      Max
-3.6052  -0.0516   0.0856   0.5237   1.2519

Coefficients:
                                Estimate Std. Error t value Pr(>|t|)
(Intercept)                   -5.3102186  2.4871692  -2.135  0.05091 .
breed_num                      1.7566699  0.6234157   2.818  0.01369 *
nr_animals.guarded             0.0775910  0.0344739   2.251  0.04100 *
observation.direct_camera_num  6.0649974  1.7059540   3.555  0.00317 **
size_guarded_area             -0.0010648  0.0003129  -3.403  0.00429 **
---
Signif. codes:  0 '***' 0.001 '**' 0.01 '*' 0.05 '.' 0.1 ' ' 1

(Dispersion parameter for gaussian family taken to be 1.515602)

    Null deviance: 72.402  on 18  degrees of freedom
Residual deviance: 21.218  on 14  degrees of freedom
AIC: 68.018

Number of Fisher Scoring iterations: 2
```

```
Call:
glm(formula = anxious.h ~ age_status_num + breed_num + farm +
    intact_num + nr_animals.guarded + observation.direct_camera_num +
    sex_num + size_group + size_guarded_area + socialisation_num,
    family = gaussian(identity), data = Dataset)

Deviance Residuals:
     Min        1Q    Median        3Q       Max
-0.50802  -0.08378   0.00000   0.16103   0.42100

Coefficients: (1 not defined because of singularities)
                                Estimate Std. Error t value Pr(>|t|)
(Intercept)                     81.65927  139.70897   0.584    0.573
age_status_num                   0.56201    0.27692   2.030    0.073 .
breed_num                     -229.61332  388.29507  -0.591    0.569
farm                           288.43449  487.40859   0.592    0.569
intact_num                      -0.32331    0.24815  -1.303    0.225
nr_animals.guarded              -3.77115    6.37744  -0.591    0.569
observation.direct_camera_num -131.79406  223.37816  -0.590    0.570
sex_num                         -0.19529    0.20261  -0.964    0.360
size_group                     -55.59140   94.11329  -0.591    0.569
size_guarded_area                0.06238    0.10552   0.591    0.569
socialisation_num                     NA         NA      NA       NA
---
Signif. codes:  0 '***' 0.001 '**' 0.01 '*' 0.05 '.' 0.1 ' ' 1

(Dispersion parameter for gaussian family taken to be 0.1264489)

    Null deviance: 3.8302  on 18  degrees of freedom
Residual deviance: 1.1380  on  9  degrees of freedom
AIC: 22.432

Number of Fisher Scoring iterations: 2
```

```
Call:
glm(formula = anxious.h ~ age_status_num + breed_num + farm,
    family = gaussian(identity), data = Dataset)

Deviance Residuals:
     Min        1Q    Median        3Q       Max
-0.55925  -0.20275   0.01218   0.15082   0.76451

Coefficients:
               Estimate Std. Error t value Pr(>|t|)
(Intercept)    -0.82039    0.40221  -2.040  0.05940 .
age_status_num  0.39052    0.21140   1.847  0.08452 .
breed_num      -0.14713    0.03735  -3.939  0.00131 **
farm            0.41848    0.10880   3.846  0.00159 **
---
Signif. codes:  0 '***' 0.001 '**' 0.01 '*' 0.05 '.' 0.1 ' ' 1

(Dispersion parameter for gaussian family taken to be 0.1153556)

    Null deviance: 3.8302  on 18  degrees of freedom
Residual deviance: 1.7303  on 15  degrees of freedom
AIC: 18.393

Number of Fisher Scoring iterations: 2
```

```
Call:
glm(formula = dominant.agressive.h ~ age_status_num + breed_num +
    farm + intact_num + nr_animals.guarded + observation.direct_camera_num +
    sex_num + size_guarded_area + size_group + socialisation_num,
    family = gaussian(identity), data = Dataset)

Deviance Residuals:
    Min       1Q   Median       3Q      Max
-3.1300  -0.6924   0.0000   0.4823   3.9891

Coefficients: (1 not defined because of singularities)
                                Estimate Std. Error t value Pr(>|t|)
(Intercept)                    165.38403  762.34241   0.217    0.833
age_status_num                   3.38258    1.51103   2.239    0.052 .
breed_num                     -484.56228 2118.78882  -0.229    0.824
farm                           610.25733 2659.61623   0.229    0.824
intact_num                       0.96136    1.35407   0.710    0.496
nr_animals.guarded              -7.92767   34.79945  -0.228    0.825
observation.direct_camera_num -274.23296 1218.89556  -0.225    0.827
sex_num                          0.07257    1.10559   0.066    0.949
size_guarded_area                0.13132    0.57579   0.228    0.825
size_group                    -117.68476  513.54295  -0.229    0.824
socialisation_num                     NA         NA      NA       NA
---
Signif. codes:  0 '***' 0.001 '**' 0.01 '*' 0.05 '.' 0.1 ' ' 1

(Dispersion parameter for gaussian family taken to be 3.765013)

    Null deviance: 82.176  on 18  degrees of freedom
Residual deviance: 33.885  on  9  degrees of freedom
AIC: 86.912

Number of Fisher Scoring iterations: 2
```

```
Call:
glm(formula = dominant.agressive.h ~ age_status_num + socialisation_num,
    family = gaussian(identity), data = Dataset)

Deviance Residuals:
    Min       1Q   Median       3Q      Max
-3.4117  -0.3245  -0.0593   0.0183   5.4541

Coefficients:
                  Estimate Std. Error t value Pr(>|t|)
(Intercept)         2.6088     0.7771   3.357  0.00401 **
age_status_num      2.7615     0.9518   2.901  0.01041 *
socialisation_num  -1.7768     0.5055  -3.515  0.00287 **
---
Signif. codes:  0 '***' 0.001 '**' 0.01 '*' 0.05 '.' 0.1 ' ' 1

(Dispersion parameter for gaussian family taken to be 2.787266)

    Null deviance: 82.176  on 18  degrees of freedom
Residual deviance: 44.596  on 16  degrees of freedom
AIC: 78.131

Number of Fisher Scoring iterations: 2
```

Principal Component Analysis directed behaviour field observation

```
Rcmdr>  Dataset <-
Rcmdr+    readXL("C:/Users/konstanze.krueger/Desktop/Ms Herdenschutz/Behaviour data guard dogs.xlsx",
Rcmdr+     rownames=FALSE, header=TRUE, na="", sheet="4 horse farms",
Rcmdr+    stringsAsFactors=TRUE)
RcmdrMsg: [2] HINWEIS: Die Datenmatrix 'Dataset' hat 15 Zeilen und 63 Spalten.

Rcmdr>  local({
Rcmdr+    .PC <-
Rcmdr+    princomp(~dominating.behaviour.category.guarded.species+dominating.behaviour.category.others+dominating.beha
viour.category.own.species,
Rcmdr+     cor=TRUE, data=Dataset)
Rcmdr+    cat("\nComponent loadings:\n")
Rcmdr+    print(unclass(loadings(.PC)))
Rcmdr+    cat("\nComponent variances:\n")
Rcmdr+    print(.PC$sd^2)
Rcmdr+    cat("\n")
Rcmdr+    print(summary(.PC))
Rcmdr+  })

Component loadings:
                                                   Comp.1     Comp.2    Comp.3
dominating.behaviour.category.guarded.species  0.04525317  0.8529188 0.5200785
dominating.behaviour.category.others           0.69074177 -0.4028180 0.6005110
dominating.behaviour.category.own.species     -0.72168411 -0.3320649 0.6073755

Component variances:
  Comp.1   Comp.2   Comp.3
1.296702 1.174282 0.529016

Importance of components:
                         Comp.1    Comp.2    Comp.3
Standard deviation     1.138728 1.0836429 0.7273349
Proportion of Variance 0.432234 0.3914273 0.1763387
Cumulative Proportion  0.432234 0.8236613 1.0000000
```

Median comparisons directed behaviour guard dogs

```
Medians:
dominating.behaviour.category.guarded.species
                                         2.25
         dominating.behaviour.category.others
                                         4.00
    dominating.behaviour.category.own.species
                                         1.25

        Friedman rank sum test

data:  .Responses
Friedman chi-squared = 4.6667, df = 2, p-value = 0.09697


        Wilcoxon signed rank test with continuity correction

data:  dominating.behaviour.category.guarded.species and dominating.behaviour.category.others
V = 15.5, p-value = 0.4376
alternative hypothesis: true location shift is not equal to 0


        Wilcoxon signed rank test with continuity correction

data:  dominating.behaviour.category.guarded.species and dominating.behaviour.category.own.species
V = 40, p-value = 0.04016
alternative hypothesis: true location shift is not equal to 0


        Wilcoxon signed rank test with continuity correction

data:  dominating.behaviour.category.others and dominating.behaviour.category.own.species
V = 71, p-value = 0.01294
alternative hypothesis: true location shift is not equal to 0
```

Generalized Linear Models behaviour directed

```
Call:
glm(formula = dominating.behaviour.category.guarded.species ~ 
    age_status_num + breed_num + farm + intact_num + nr_animals.guarded + 
        observation.direct_camera_num + sex_num + size_group + 
        size_guarded_area, family = poisson(identity), data = Dataset)

Deviance Residuals: 
       1         2         3         4         5         6         7         8  
 0.00000             0.00000            -0.70125   0.12505  -0.49762   0.00000  
       9        10        11        12        13        14        15  
 0.82125             0.71972  -1.15786  -0.08495   0.19243   0.19512  

Coefficients: (2 not defined because of singularities)
                              Estimate Std. Error z value Pr(>|z|)
(Intercept)                   -1.26997    3.30122  -0.385    0.700
age_status_num                -0.91470    1.73314  -0.528    0.598
breed_num                     -0.04822    0.70299  -0.069    0.945
farm                          -0.47841    0.89282  -0.536    0.592
intact_num                    -1.12611    2.14372  -0.525    0.599
nr_animals.guarded             0.47337    0.65632   0.721    0.471
observation.direct_camera_num       NA         NA      NA       NA
sex_num                        0.99568    1.41510   0.704    0.482
size_group                     0.43769    0.45163   0.969    0.332
size_guarded_area                   NA         NA      NA       NA

(Dispersion parameter for poisson family taken to be 1)

    Null deviance: 6.2707  on 11  degrees of freedom
Residual deviance: 3.3704  on  4  degrees of freedom
  (3 observations deleted due to missingness)
AIC: Inf
```

```
Call:
glm(formula = dominating.behaviour.category.others ~ age_status_num +
    breed_num + farm + intact_num + nr_animals.guarded + observation.direct_camera_num +
    sex_num + size_group + size_guarded_area, family = poisson(identity),
    data = Dataset)

Deviance Residuals:
     Min        1Q    Median        3Q       Max
-1.20853  -0.18853   0.01736   0.17728   0.71362

Coefficients: (2 not defined because of singularities)
                              Estimate Std. Error z value Pr(>|z|)
(Intercept)                     3.5327     4.6361   0.762    0.446
age_status_num                  2.2759     1.6502   1.379    0.168
breed_num                       0.1528     0.7491   0.204    0.838
farm                            1.2871     0.8652   1.488    0.137
intact_num                     -1.1039     1.9464  -0.567    0.571
nr_animals.guarded             -0.5081     0.7595  -0.669    0.503
observation.direct_camera_num       NA         NA      NA       NA
sex_num                        -1.7615     1.2019  -1.466    0.143
size_group                     -0.3441     0.4994  -0.689    0.491
size_guarded_area                   NA         NA      NA       NA

(Dispersion parameter for poisson family taken to be 1)

    Null deviance: 7.4868  on 13  degrees of freedom
Residual deviance: 2.6398  on  6  degrees of freedom
  (1 observation deleted due to missingness)
AIC: Inf
```

```
Call:
glm(formula = dominating.behaviour.category.own.species ~ age_status_num + 
    breed_num + farm + intact_num + nr_animals.guarded + observation.direct_camera_num + 
    sex_num + size_group + size_guarded_area, family = poisson(identity), 
    data = Dataset)

Deviance Residuals: 
     Min        1Q    Median        3Q       Max  
-0.44215  -0.17533  -0.09287   0.15101   0.74297  

Coefficients: (2 not defined because of singularities)
                              Estimate Std. Error z value Pr(>|z|)
(Intercept)                    2.11627    3.24785   0.652    0.515
age_status_num                -0.50746    1.39470  -0.364    0.716
breed_num                     -0.26635    0.63872  -0.417    0.677
farm                           0.12669    0.71133   0.178    0.859
intact_num                    -0.58099    1.43343  -0.405    0.685
nr_animals.guarded             0.04721    0.52672   0.090    0.929
observation.direct_camera_num       NA         NA      NA       NA
sex_num                       -0.43377    1.15032  -0.377    0.706
size_group                     0.09069    0.33539   0.270    0.787
size_guarded_area                   NA         NA      NA       NA

(Dispersion parameter for poisson family taken to be 1)

    Null deviance: 4.5990  on 13  degrees of freedom
Residual deviance: 1.3201  on  6  degrees of freedom
  (1 observation deleted due to missingness)
AIC: Inf
```

b) statistical analyses of the protocols.

Principal Component Analysis protocol data

```
Rcmdr>  local({
Rcmdr+    .PC <-
Rcmdr+    princomp(~behav..to.dogs+behav..to.farm.animals+behav..to.known.humans.no.owner+behav..to.known.humans.with.owner+behav..
to.owner+behav..to.unknown.humans.no.owner+behav..to.unknown.humans.with.owner,
Rcmdr+    cor=TRUE, data=Dataset)
Rcmdr+    cat("\nComponent loadings:\n")
Rcmdr+    print(unclass(loadings(.PC)))
Rcmdr+    cat("\nComponent variances:\n")
Rcmdr+    print(.PC$sd^2)
Rcmdr+    cat("\n")
Rcmdr+    print(summary(.PC))
Rcmdr+  })

Component loadings:
                                      Comp.1      Comp.2       Comp.3      Comp.4      Comp.5      Comp.6       Comp.7
behav..to.dogs                     0.3801018  0.28884210  0.457357430  0.58793291  0.07321820  0.45315832  0.080861339
behav..to.farm.animals             0.2319199  0.42369315  0.518520335 -0.41883704 -0.35917046 -0.33580921 -0.283968703
behav..to.known.humans.no.owner    0.3759518 -0.52887286  0.004219381  0.05178609  0.09306233  0.06914421 -0.750208391
behav..to.known.humans.with.owner  0.3760066 -0.46919824  0.170755217  0.23839487 -0.19408355 -0.54902516  0.461936725
behav..to.owner                    0.3882263  0.17878228 -0.596286955  0.05986342 -0.65007895  0.18851120  0.006027682
behav..to.unknown.humans.no.owner -0.2837409 -0.44961716  0.367717801 -0.22841758 -0.50356221  0.50526532  0.145177800
behav..to.unknown.humans.with.owner  0.5364573 -0.05551739 -0.044846199 -0.60302429  0.37832408  0.29127416  0.339870748

Component variances:
   Comp.1    Comp.2    Comp.3    Comp.4    Comp.5    Comp.6    Comp.7
2.4201194 2.2926660 1.0107674 0.5705370 0.4549035 0.2331232 0.0178834

Importance of components:
                          Comp.1    Comp.2    Comp.3     Comp.4     Comp.5     Comp.6      Comp.7
Standard deviation     1.5556733 1.5141552 1.0053693 0.75533902 0.67446537 0.48282830 0.133728834
Proportion of Variance 0.3457313 0.3275237 0.1443953 0.08150529 0.06498622 0.03330331 0.002554772
Cumulative Proportion  0.3457313 0.6732551 0.8176504 0.89915570 0.96414192 0.99744523 1.000000000
```

Median comparisons protocol data

```
Medians:
                  to.dogs              to.farm.animals    to.known.person.no.owner  to.known.person.with.owner                  to.owner
                      4.0                          2.0                         1.5                         1.0                       1.0
  to.unknown.person.no.owner to.unknown.person.with.owner
                      4.0                          1.5
```

Friedman rank sum test

```
data:  .Responses
Friedman chi-squared = 37.52, df = 6, p-value = 0.000001394
```

Factor Analysis protocol data

```
Rcmdr>  local({
Rcmdr+    .FA <-
Rcmdr+    factanal(~age.group...11+animals.at.farm+breeds...7+duration.owner.has.guard.dogs+for.wolf.protection+groups
..animals.protected+intact+location+number.guard.dogs+observer+sex+socialisation+species.at.farm+task.change+tasks,
Rcmdr+     factors=3, rotation="varimax", scores="none", data=Dataset)
Rcmdr+    print(.FA)
Rcmdr+  })

Call:
factanal(x = ~age.group...11 + animals.at.farm + breeds...7 +     duration.owner.has.guard.dogs + for.wolf.protection
+ groups..animals.protected +     intact + location + number.guard.dogs + observer + sex +     socialisation + species
.at.farm + task.change + tasks, factors = 3,     data = Dataset, scores = "none", rotation = "varimax")

Uniquenesses:
              age.group...11               animals.at.farm                     breeds...7 duration.owner.has.guard.dog
s
                       0.005                         0.815                          0.616                         0.00
5
         for.wolf.protection     groups..animals.protected                         intact                     locatio
n
                       0.640                         0.337                          0.635                         0.64
7
           number.guard.dogs                      observer                            sex                  socialisatio
n
                       0.327                         0.923                          0.276                         0.53
0
             species.at.farm                   task.change                          tasks
                       0.291                         0.393                          0.823
```

```
Loadings:
                              Factor1 Factor2 Factor3
age.group...11                 0.817   0.181   0.543 
animals.at.farm                                0.425 
breeds...7                     0.174   0.561   0.198 
duration.owner.has.guard.dogs  0.173   0.597   0.780 
for.wolf.protection            0.299   0.515         
groups..animals.protected      0.798   0.112  -0.116 
intact                                 0.590  -0.100 
location                      -0.593                 
number.guard.dogs              0.408   0.711         
observer                      -0.141   0.234         
sex                                    0.804   0.277 
socialisation                         -0.681         
species.at.farm                0.715   0.288  -0.339 
task.change                    0.758   0.174         
tasks                          0.417                 

               Factor1 Factor2 Factor3
SS loadings      3.266   3.118   1.352
Proportion Var   0.218   0.208   0.090
Cumulative Var   0.218   0.426   0.516

Test of the hypothesis that 3 factors are sufficient.
The chi square statistic is 72.37 on 63 degrees of freedom.
The p-value is 0.196
```

Generalized Linear Models behaviour directed

```
Call:
glm(formula = to.alien.person ~ age.group...11 + breeds...7 +
    intact + location + observer + sex + socialisation + animals.at.farm +
    duration.owner.has.guard.dogs + for.wolf.protection + groups..animals.protected +
    number.guard.dogs + species.at.farm + task.change + tasks,
    family = poisson(identity), data = Dataset)

Coefficients:
                                 Estimate  Std. Error z value Pr(>|z|)
(Intercept)                    6.11372245  9.84478840   0.621    0.535
age.group...11                -1.20249845  3.57435587  -0.336    0.737
breeds...7                     0.13937793  0.35924377   0.388    0.698
intact                        -0.49275144  0.85540285  -0.576    0.565
location                       0.40347102  1.37961974   0.292    0.770
observer                      -0.12115418  0.35801821  -0.338    0.735
sex                           -0.79314975  2.50688283  -0.316    0.752
socialisation                 -0.48189117  0.77496703  -0.622    0.534
animals.at.farm                0.00005875  0.00096431   0.061    0.951
duration.owner.has.guard.dogs  1.09106930  5.11825115   0.213    0.831
for.wolf.protection           -0.69124348  3.24614675  -0.213    0.831
groups..animals.protected     -0.14379930  2.15360692  -0.067    0.947
number.guard.dogs              0.10223190  0.55638899   0.184    0.854
species.at.farm                0.10295114  0.81558342   0.126    0.900
task.change                    1.73113292  2.73651733   0.633    0.527
tasks                          0.36496477  2.42293741   0.151    0.880

(Dispersion parameter for poisson family taken to be 1)

    Null deviance: 3.76714  on 18  degrees of freedom
Residual deviance: 0.41021  on  3  degrees of freedom
  (6 Beobachtungen als fehlend gelöscht)
AIC: 91.276

Number of Fisher Scoring iterations: 5
```

```
Call:
glm(formula = to.alien.person ~ age.group...11 + groups..animals.protected +
    task.change + duration.owner.has.guard.dogs, family = poisson(identity),
    data = Dataset)

Coefficients:
                               Estimate Std. Error z value Pr(>|z|)
(Intercept)                    4.546701   1.902922   2.389   0.0169 *
age.group...11                -1.310082   0.770099  -1.701   0.0889 .
groups..animals.protected      0.009972   0.669852   0.015   0.9881
task.change                    1.859906   1.851660   1.004   0.3152
duration.owner.has.guard.dogs  0.981008   0.734504   1.336   0.1817
---
Signif. codes:  0 '***' 0.001 '**' 0.01 '*' 0.05 '.' 0.1 ' ' 1

(Dispersion parameter for poisson family taken to be 1)

    Null deviance: 3.7671  on 18  degrees of freedom
Residual deviance: 1.5409  on 14  degrees of freedom
  (6 Beobachtungen als fehlend gelöscht)
AIC: 70.407
```

```
Call:
glm(formula = to.alien.person.owner ~ age.group...11 + breeds...7 +
    intact + location + observer + sex + socialisation + animals.at.farm +
    duration.owner.has.guard.dogs + for.wolf.protection + groups..animals.protected +
    number.guard.dogs + species.at.farm + task.change + tasks,
    family = poisson(identity), data = Dataset)

Coefficients:
                                Estimate Std. Error z value Pr(>|z|)
(Intercept)                    6.0682488  7.3113371   0.830    0.407
age.group...11                 1.9684689  2.6414169   0.745    0.456
breeds...7                     0.2313389  0.2528442   0.915    0.360
intact                        -0.2918162  0.6645971  -0.439    0.661
location                      -0.8337243  0.9638416  -0.865    0.387
observer                       0.2389791  0.2290319   1.043    0.297
sex                            1.2839257  1.6600290   0.773    0.439
socialisation                  0.0145235  0.6016773   0.024    0.981
animals.at.farm                0.0007376  0.0007502   0.983    0.325
duration.owner.has.guard.dogs -3.2372029  3.8643249  -0.838    0.402
for.wolf.protection            2.2889579  2.2991226   0.996    0.319
groups..animals.protected     -1.6131508  1.5989619  -1.009    0.313
number.guard.dogs              0.0758729  0.4293864   0.177    0.860
species.at.farm               -0.2105118  0.5305199  -0.397    0.692
task.change                    1.5012865  2.0183637   0.744    0.457
tasks                         -2.3739999  1.7544884  -1.353    0.176

(Dispersion parameter for poisson family taken to be 1)

    Null deviance: 6.20415  on 18  degrees of freedom
Residual deviance: 0.11897  on  3  degrees of freedom
  (6 Beobachtungen als fehlend gelöscht)
AIC: 78.382   Call:
```

```
glm(formula = to.alien.person.owner ~ duration.owner.has.guard.dogs +
    animals.at.farm + task.change + groups..animals.protected,
    family = poisson(identity), data = Dataset)

Coefficients:
                                Estimate Std. Error z value Pr(>|z|)
(Intercept)                    1.0258690  1.2142296   0.845    0.398
duration.owner.has.guard.dogs  0.1875835  0.4271776   0.439    0.661
animals.at.farm                0.0002023  0.0002705   0.748    0.454
task.change                   -0.1645531  1.2007445  -0.137    0.891
groups..animals.protected      0.0775308  0.4322017   0.179    0.858

(Dispersion parameter for poisson family taken to be 1)

    Null deviance: 6.2042  on 18  degrees of freedom
Residual deviance: 5.0206  on 14  degrees of freedom
  (6 Beobachtungen als fehlend gelöscht)
AIC: 61.284
```

```
Call:
glm(formula = to.dogs ~ age.group...11 + breeds...7 + intact +
    location + observer + sex + socialisation + animals.at.farm +
    duration.owner.has.guard.dogs + for.wolf.protection + groups..animals.protected +
    number.guard.dogs + species.at.farm + task.change + tasks,
    family = poisson(identity), data = Dataset)

Coefficients: (1 not defined because of singularities)
                               Estimate Std. Error z value Pr(>|z|)
(Intercept)                    6.922968  30.291514   0.229    0.819
age.group...11                -2.485598  15.680172  -0.159    0.874
breeds...7                    -0.033306   0.825811  -0.040    0.968
intact                        -0.244224   2.574644  -0.095    0.924
location                      -2.486354   7.585253  -0.328    0.743
observer                       0.510100   1.630751   0.313    0.754
sex                            1.704110   8.430304   0.202    0.840
socialisation                 -0.272083   2.510328  -0.108    0.914
animals.at.farm               -0.002166   0.006349  -0.341    0.733
duration.owner.has.guard.dogs  1.524529  21.372271   0.071    0.943
for.wolf.protection           -0.348832   9.804741  -0.036    0.972
groups..animals.protected     -0.301475   7.082188  -0.043    0.966
number.guard.dogs              0.265100   1.693514   0.157    0.876
species.at.farm                0.469641   2.115953   0.222    0.824
task.change                    1.727225   4.441295   0.389    0.697
tasks                                NA         NA      NA       NA

(Dispersion parameter for poisson family taken to be 1)

    Null deviance: 5.7826e+00  on 14  degrees of freedom
Residual deviance: 1.7764e-15  on  0  degrees of freedom
  (10 Beobachtungen als fehlend gelöscht)
AIC: 75.359
```

```
Call:
glm(formula = to.dogs ~ sex + groups..animals.protected, family = poisson(identity),
    data = Dataset)

Coefficients:
                          Estimate Std. Error z value Pr(>|z|)
(Intercept)               -0.55324    1.60011  -0.346  0.72953
sex                        1.41257    0.53399   2.645  0.00816 **
groups..animals.protected  0.01419    0.44424   0.032  0.97452
---
Signif. codes:  0 '***' 0.001 '**' 0.01 '*' 0.05 '.' 0.1 ' ' 1

(Dispersion parameter for poisson family taken to be 1)

    Null deviance: 5.7826  on 14  degrees of freedom
Residual deviance: 1.7757  on 12  degrees of freedom
  (10 Beobachtungen als fehlend gelöscht)
AIC: 53.135
```

```
Call:
glm(formula = to.farm.animals ~ age.group...11 + breeds...7 +
    intact + location + observer + sex + socialisation + animals.at.farm +
    duration.owner.has.guard.dogs + for.wolf.protection + groups..animals.protected +
    number.guard.dogs + species.at.farm + tasks + task.change,
    family = poisson(identity), data = Dataset)

Coefficients: (2 not defined because of singularities)
                                Estimate Std. Error z value Pr(>|z|)
(Intercept)                   -6.5393660 10.6599476  -0.613    0.540
age.group...11                 1.1071535  5.3538978   0.207    0.836
breeds...7                     0.8601728  0.7060269   1.218    0.223
intact                        -1.0041761  1.4461585  -0.694    0.487
location                       2.9605811  3.6343006   0.815    0.415
observer                      -0.3881493  1.0987959  -0.353    0.724
sex                           -0.0450576  4.4762807  -0.010    0.992
socialisation                 -0.5253640  0.8180659  -0.642    0.521
animals.at.farm               -0.0002799  0.0005411  -0.517    0.605
duration.owner.has.guard.dogs -1.7429125  8.7117348  -0.200    0.841
for.wolf.protection           -5.4206135  4.9148954  -1.103    0.270
groups..animals.protected      1.3048175  4.0272004   0.324    0.746
number.guard.dogs              1.4051598  1.1800810   1.191    0.234
species.at.farm               -0.7295974  0.6529073  -1.117    0.264
tasks                                 NA         NA      NA       NA
task.change                           NA         NA      NA       NA

(Dispersion parameter for poisson family taken to be 1)

    Null deviance: 4.9001e+00  on 13  degrees of freedom
Residual deviance: 8.8818e-16  on  0  degrees of freedom
  (11 Beobachtungen als fehlend gelöscht)
AIC: 62.554

Number of Fisher Scoring iterations: 2
```

```
Call:
glm(formula = to.farm.animals ~ duration.owner.has.guard.dogs, 
    family = poisson(identity), data = Dataset)

Coefficients:
                              Estimate Std. Error z value Pr(>|z|)
(Intercept)                     1.2199     1.3659   0.893    0.372
duration.owner.has.guard.dogs   0.2348     0.4976   0.472    0.637

(Dispersion parameter for poisson family taken to be 1)

    Null deviance: 4.9001  on 13  degrees of freedom
Residual deviance: 4.7155  on 12  degrees of freedom
  (11 Beobachtungen als fehlend gelöscht)
AIC: 43.269
```

```
Call:
glm(formula = to.known.person ~ age.group...11 + breeds...7 +
    intact + location + observer + sex + socialisation + animals.at.farm +
    duration.owner.has.guard.dogs + for.wolf.protection + groups..animals.protected +
    number.guard.dogs + species.at.farm + task.change + tasks,
    family = poisson(identity), data = Dataset)

Coefficients: (4 not defined because of singularities)
                               Estimate Std. Error z value Pr(>|z|)
(Intercept)                    5.069817  13.499127   0.376    0.707
age.group...11                 2.359853   2.128253   1.109    0.268
breeds...7                     0.036875   0.562996   0.065    0.948
intact                        -0.915461   0.920997  -0.994    0.320
location                      -0.197147   4.023794  -0.049    0.961
observer                      -0.177171   0.696384  -0.254    0.799
sex                            1.201687   2.803736   0.429    0.668
socialisation                 -0.290193   0.439836  -0.660    0.509
animals.at.farm                0.002330   0.001491   1.563    0.118
duration.owner.has.guard.dogs -3.677558   3.915374  -0.939    0.348
for.wolf.protection            1.144308   2.550143   0.449    0.654
groups..animals.protected     -2.062629   2.154292  -0.957    0.338
number.guard.dogs                    NA         NA      NA       NA
species.at.farm                      NA         NA      NA       NA
task.change                          NA         NA      NA       NA
tasks                                NA         NA      NA       NA

(Dispersion parameter for poisson family taken to be 1)

    Null deviance: 5.2685e+00  on 11  degrees of freedom
Residual deviance: 4.4409e-16  on  0  degrees of freedom
  (13 Beobachtungen als fehlend gelöscht)
AIC: 52.712
```

```
Call:
glm(formula = to.known.person ~ animals.at.farm + duration.owner.has.guard.dogs +
    groups..animals.protected, family = poisson(identity), data = Dataset)

Coefficients:
                               Estimate Std. Error z value Pr(>|z|)
(Intercept)                   2.8235081  1.7038504   1.657   0.0975 .
animals.at.farm               0.0008968  0.0006600   1.359   0.1742
duration.owner.has.guard.dogs -0.3957816  0.5161770  -0.767   0.4432
groups..animals.protected     -0.5427980  0.6921205  -0.784   0.4329
---
Signif. codes:  0 '***' 0.001 '**' 0.01 '*' 0.05 '.' 0.1 ' ' 1

(Dispersion parameter for poisson family taken to be 1)

    Null deviance: 5.2685  on 11  degrees of freedom
Residual deviance: 2.8554  on  8  degrees of freedom
  (13 Beobachtungen als fehlend gelöscht)
AIC: 39.568
```

```
Call:
glm(formula = to.known.person.owner ~ age.group...11 + breeds...7 +
    intact + location + observer + sex + socialisation + animals.at.farm +
    duration.owner.has.guard.dogs + for.wolf.protection + groups..animals.protected +
    number.guard.dogs + species.at.farm + task.change + tasks,
    family = poisson(identity), data = Dataset)

Coefficients: (3 not defined because of singularities)
                                Estimate Std. Error z value Pr(>|z|)
(Intercept)                   1072.79832  819.68602   1.309    0.191
age.group...11                 -20.64000   17.37710  -1.188    0.235
breeds...7                     -66.09414   50.53516  -1.308    0.191
intact                         110.99623   84.78039   1.309    0.190
location                      -379.22701  290.03725  -1.308    0.191
observer                        72.57153   55.53025   1.307    0.191
sex                             83.91344   63.44303   1.323    0.186
socialisation                   36.72013   28.03135   1.310    0.190
animals.at.farm                 -0.09694    0.07515  -1.290    0.197
duration.owner.has.guard.dogs  -47.89709   34.72826  -1.379    0.168
for.wolf.protection            427.80134  326.26134   1.311    0.190
groups..animals.protected     -141.51085  107.28722  -1.319    0.187
number.guard.dogs              -89.13068   68.01659  -1.310    0.190
species.at.farm                       NA         NA      NA       NA
task.change                           NA         NA      NA       NA
tasks                                 NA         NA      NA       NA

(Dispersion parameter for poisson family taken to be 1)

    Null deviance: 6.3464e+00  on 12  degrees of freedom
Residual deviance: 4.4409e-16  on  0  degrees of freedom
  (12 Beobachtungen als fehlend gelöscht)
AIC: 57.091
```

```
Call:
glm(formula = to.known.person.owner ~ breeds...7 + intact + location +
    observer + socialisation + for.wolf.protection + number.guard.dogs +
    groups..animals.protected + duration.owner.has.guard.dogs +
    task.change + tasks, family = poisson(identity), data = Dataset)

Coefficients:
                              Estimate Std. Error z value Pr(>|z|)
(Intercept)                    25.5749    16.7633   1.526   0.1271
breeds...7                     -4.8777     2.8681  -1.701   0.0890 .
intact                         11.1594     6.4657   1.726   0.0844 .
location                      -17.0763     9.7466  -1.752   0.0798 .
observer                        1.6278     0.8842   1.841   0.0656 .
socialisation                   4.7303     2.8732   1.646   0.0997 .
for.wolf.protection            24.0919    13.7360   1.754   0.0794 .
number.guard.dogs              -8.3725     4.8883  -1.713   0.0868 .
groups..animals.protected      -1.4482     2.1565  -0.672   0.5019
duration.owner.has.guard.dogs   5.4772     3.9794   1.376   0.1687
task.change                   -23.4375    16.0383  -1.461   0.1439
tasks                          13.3351     8.7659   1.521   0.1282
---
Signif. codes:  0 '***' 0.001 '**' 0.01 '*' 0.05 '.' 0.1 ' ' 1

(Dispersion parameter for poisson family taken to be 1)

    Null deviance: 6.346405  on 12  degrees of freedom
Residual deviance: 0.059799  on  1  degrees of freedom
  (12 Beobachtungen als fehlend gelöscht)
AIC: 55.15
```

```
Call:
glm(formula = to.owner ~ age.group...11 + breeds...7 + intact + 
    location + observer + sex + socialisation + animals.at.farm + 
    duration.owner.has.guard.dogs + for.wolf.protection + groups..animals.protected + 
    number.guard.dogs + species.at.farm + task.change + tasks, 
    family = poisson(identity), data = Dataset)

Coefficients:
                                Estimate Std. Error z value Pr(>|z|)
(Intercept)                   -1.4522340  5.3607510  -0.271    0.786
age.group...11                 1.0320058  2.0216137   0.510    0.610
breeds...7                     0.0802419  0.1959999   0.409    0.682
intact                         0.3344189  0.5207213   0.642    0.521
location                       0.2956950  0.7496915   0.394    0.693
observer                       0.0919244  0.1922794   0.478    0.633
sex                            0.7237059  1.3771427   0.526    0.599
socialisation                  0.0965876  0.4086558   0.236    0.813
animals.at.farm                0.0001291  0.0004990   0.259    0.796
duration.owner.has.guard.dogs -1.5190538  2.8402870  -0.535    0.593
for.wolf.protection            0.1727381  1.6675110   0.104    0.917
groups..animals.protected     -0.1254323  1.1394209  -0.110    0.912
number.guard.dogs             -0.0947945  0.3150590  -0.301    0.764
species.at.farm               -0.0912017  0.4595121  -0.198    0.843
task.change                   -0.6854450  1.5262209  -0.449    0.653
tasks                         -0.2203611  1.2229162  -0.180    0.857

(Dispersion parameter for poisson family taken to be 1)

    Null deviance: 1.86721  on 18  degrees of freedom
Residual deviance: 0.20974  on  3  degrees of freedom
  (6 Beobachtungen als fehlend gelöscht)
AIC: 72.051
```

```
Call:
glm(formula = to.owner ~ groups..animals.protected + animals.at.farm +
    duration.owner.has.guard.dogs + task.change, family = poisson(identity),
    data = Dataset)

Coefficients:
                                Estimate  Std. Error z value Pr(>|z|)
(Intercept)                   1.37932848  1.05560981   1.307    0.191
groups..animals.protected     0.07070624  0.34723608   0.204    0.839
animals.at.farm               0.00003251  0.00018994   0.171    0.864
duration.owner.has.guard.dogs -0.12934976  0.36581652  -0.354    0.724
task.change                  -0.25600255  0.97172619  -0.263    0.792

(Dispersion parameter for poisson family taken to be 1)

    Null deviance: 1.8672  on 18  degrees of freedom
Residual deviance: 1.5639  on 14  degrees of freedom
  (6 Beobachtungen als fehlend gelöscht)
AIC: 51.405
```
